# A consensus-based template for documenting and reporting in physician-staffed pre-hospital services

**DOI:** 10.1186/1757-7241-19-71

**Published:** 2011-11-23

**Authors:** Andreas J Krüger, David Lockey, Jouni Kurola, Stefano Di Bartolomeo, Maaret Castrén, Søren Mikkelsen, Hans Morten Lossius

**Affiliations:** 1 Department of Research and Development, Norwegian Air Ambulance Foundation, Drøbak, Norway; 2 Department of Anaesthesia and Emergency Medicine, St. Olav University Hospital, Trondheim, Norway; 3 Department of Circulation and Medical Imaging, Faculty of Medicine, Norwegian University of Science and Technology (NTNU), Trondheim, Norway; 4 North Bristol NHS trust, Bristol, UK; 5 Centre for Prehospital Care, division of prehospital emergency care, emergency and intensive care, Kuopio University Hospital, Kuopio, Finland; 6 Anaesthesia and ICU S.M.M Hospital, Udine/Regional Health Agency of Emilia-Romagna, Bologna, Italy; 7 Karolinska Institutet, Department of Clinical Science and Education, Södersjukhuset and Section of Emergency Medicine, Södersjukhuset, Stockholm, Sweden; 8 Mobile Emergency Care Unit, Department of Anaesthesiology and Intensive Care Medicine, Odense University Hospital, Odense, Denmark; 9 Department of Surgical Sciences, University of Bergen, Norway

## Abstract

**Background:**

Physician-staffed pre-hospital units are employed in many Western emergency medical services (EMS) systems. Although these services usually integrate well within their EMS, little is known about the quality of care delivered, the precision of dispatch, and whether the services deliver a higher quality of care to pre-hospital patients. There is no common data set collected to document the activity of physician pre-hospital activity which makes shared research efforts difficult. The aim of this study was to develop a core data set for routine documentation and reporting in physician-staffed pre-hospital services in Europe.

**Methods:**

Using predefined criteria, we recruited sixteen European experts in the field of pre-hospital care. These experts were guided through a four-step modified nominal group technique. The process was carried out using both e-mail-based communication and a plenary meeting in Stavanger, Norway.

**Results:**

The core data set was divided into 5 sections: "fixed system variables", "event operational descriptors", " patient descriptors", "process mapping", and "outcome measures and quality indicators". After the initial round, a total of 361 variables were proposed by the experts. Subsequent rounds reduced the number of core variables to 45. These constituted the final core data set. Emphasis was placed on the standardisation of reporting time variables, chief complaints and diagnostic and therapeutic procedures.

**Conclusions:**

Using a modified nominal group technique, we have established a core data set for documenting and reporting in physician-staffed pre-hospital services. We believe that this template could facilitate future studies within the field and facilitate standardised reporting and future shared research efforts in advanced pre-hospital care.

## Background

Pre-hospital emergency medicine aims to provide early, high-quality, goal-directed treatment. The time to "restoration of adequate flow and physiology" is recognised as crucial in several conditions, including cardiac arrest, cardiac ischaemia, severe trauma, sepsis, respiratory distress, stroke and obstetric emergencies [1]. As our knowledge of acute illness and injury evolves, rapid diagnostics and treatment are becoming more important.

It has been argued that an appropriately trained physician is able to provide superior treatment compared to other emergency medical care providers, and, based on this assumption, having a physician as an integrated part of pre-hospital emergency medical care will positively affect patient outcomes [2-5]. These services typically consist of rapid and flexible means of transportation. In larger cities, rapid response cars are often available, and helicopters are common in rural areas. These means of transportation [6,7] facilitate rapid access to the patient and can reduce the time to definitive care at appropriate medical facilities.

The role of the physician-staffed pre-hospital services is not solely medical treatment. The physician often takes an active part in patient and resource triage and acts as a medical advisor/supervisor for ambulance crews.

In Scandinavia, physician-staffed pre-hospital services are well-established and commonly manned with anaesthesiologists to deliver critical emergency care. These services are publicly funded and operate 24 hours a day. The means of transportation (helicopter, rapid response car, ambulance) are provided according to logistical needs. The majority of services are well integrated within their local emergency medical systems [6].

Physician-staffed pre-hospital services demand more resources and are more expensive than standard paramedic-staffed services. The evidence base for which patient groups benefit from physician-manned services and which system factors are related to a favourable patient outcome is currently weak [8].

More research is required to evaluate the effect and efficiency of such services[9]. The well-organised and relatively homogeneous systems of Scandinavia are suited for collaborative research projects. Unfortunately, proper documentation and reporting of medical and operational activity is uncommon and the data that is recorded lacks common variables and definitions. This lack of systematisation of documentation within and between countries is nearly generic in an international perspective and makes it difficult to compare services and to conduct systematic searches in the published literature and multicentre-based prospective or registry- based research [10-13].

In several emergency medical fields, shared research efforts based on common reporting templates have proven useful [14-17]. The Utstein Template for documenting and reporting in cardiac arrest is probably the most widely used template and has facilitated a large amount of research [18-21]. The rationale is to document and report data prospectively with a predefined template developed by experts [22-24]. These templates gather data that can be easily and routinely reported, making multicentre-based research feasible [25,26]). Considerably less agreement currently exists on the reporting of the effect and efficiency of emergency medical systems. There is no international agreement on the reporting of fundamental variables, such as outcome variables [10]. Research addressing structure, process and outcome quality indicators, and factors associated with increased value is lacking [27,28].

Templates for documenting and reporting data may be developed using multidisciplinary expert panel consensus methods [27,29,30]. Expert panel assessments allow a combination of evidence-based knowledge, personal experience, and general insight in the characteristics of the patient cohort assessed or problem addressed [31], and has been the method of choice in the development of previous Utstein templates [24,32-37]

The aim of this project was to develop a common Utstein-like template for documenting and reporting on pre-hospital emergency medical services manned with trained pre-hospital physicians.

## Methods

The current work is the third part of the ScanDoc project, a Scandinavian consortium working for better research infrastructure in advanced pre-hospital care.

We used a modified nominal group technique [31], and defined "physician-staffed pre-hospital unit" as a dedicated unit staffed with a physician with competency in critical emergency care above that of an ordinary on-call general practitioner.

### The experts

The ScanDoc representatives assisted in proposing experts from the Scandinavian countries. Several countries in central Europe operate pre-hospital physician staffed services similar to those in Scandinavia, and, therefore, it was decided to invite representatives from other countries in Europe. As the original purpose of the ScanDoc-project was to standardise reporting from the Scandinavian countries, the project group decided to weight the panel composition toward Scandinavia, resulting in three representatives from Scandinavia per one from another European country. Based on the following inclusion criteria, panel members were recruited from Sweden, Finland, Denmark, Norway, Italy, Germany, Austria, Switzerland, Holland and the United Kingdom.

1. Relevant clinical experience working in a physician pre-hospital service to ensure personal insight into the operative and medical characteristics of advanced pre-hospital care.

2. Scientific and/or substantial leadership responsibilities in pre-hospital care to ensure competency in research methods and governance of pre-hospital emergency systems.

3. Ability to communicate in English.

The experts were identified using Medline and Google Scholar searches and via the professional network of the project group. Each expert was invited into the consensus process by e-mail or telephone when feasible. Non-responders were reminded three times by e-mail, followed by direct contact from one of the members of the project group. The selected expert panel participated throughout the complete process, and no additional experts were included after initiating the process.

### The modified nominal group process

The consensus process was arranged in a four-step sequence (details of each stage are described below). Stages 1, 2 and 4 were carried out by email. During stage 3, the panel met in Stavanger, Norway for a 2-day consensus meeting.

Keeping proposals anonymous is of major methodological importance to reduce the influence of "loud-speaking" experts and to facilitate the influence of their "silent-speaking" peers [31,38]. The expert panel members were aware of its composition, but anonymity related to proposals was secured until stage 3.

Stage 1. The expert panel received a predesigned worksheet by email. Each expert was asked to propose the ten most important variables to be routinely documented for shared research and benchmarking within each of five predefined sections:

1. Fixed system variables

Variables relating to system characteristics concerning how the service is organised, the operational capacities of the service and its integration with the EMS with which it operate.

2. Event operational descriptors

Variables documenting the context of a mission (dispatch) or episodes of use (for services with advisory functions).

3. Patient descriptors

Variables documenting information related to the patient's profile, e.g., age, gender, co-morbidity and type of medical complaint.

4. Process mapping

Variables recording what happened to the patient and how the episodes of care proceeded.

5. Outcome and quality indicators

Variables relating to patient and/or mission outcomes, as well as measures of quality.

An optional sixth section for proposals of variables that did not fit into one of the predefined sections was provided. The experts were informed that a subsidiary aim of the process was to establish a core data set that was easy to routinely collect and did not require excessive database alterations.

The project organisers recognised the challenge that several reports on how to document and report data in various parts of emergency care have already been published and implemented [24,32,37,39]. These templates (e.g., Utstein for cardiac arrest, the Utstein template for major trauma and the Utstein template for the reporting of advanced airway management) contain some common variables with slightly different definitions. The expert panel was supplied these published templates and asked to make the new variables compatible with existing template variables, if feasible.

The proposed variables were returned to the project group by email and systemised. Different variables with identical meaning were combined carefully so as not to interfere with the expert panel's proposals. No single proposed variables were deleted. The variables within each section were ranked according to how many times the variables had been proposed by the different expert panel members.

Stage 2. The revised worksheet containing aggregated results from stage 1 was sent to the expert panel. The panel were then requested to rank the ten most important variables in each section from 10 (most important) to 1 (least important). The variables with no ranking were then removed from the list. The results from this ranking provided the basis for the consensus meeting.

Stage 3. The expert panel gathered in Stavanger, Norway and, during a 2-day meeting, agreed upon a core data set. At this meeting, moderators (DL and HML)) led the experts through the results from stage 2. The experts were divided into two groups and discussed portions of the preliminary dataset. The experts subsequently presented their discussions in plenum, and variables were discussed, debated and agreed upon. The variables were decided upon on day 1 and were defined and categorised on day 2. Some variables were not finally defined during the meeting, and the expert panel approved the project group to propose definitions for the remaining variables before stage 4.

Stage 4. Based on step 3, the final data set, including definitions, was prepared by the project group and submitted to the expert panel for final approval. At this point, no additional variables were accepted, but minor changes related to answer categories and definitions were allowed.

Consensus was defined as agreement on the proposed variables at the consensus meeting among the attending experts. Furthermore, we informed the experts during stage 4 that no response was interpreted as agreement to the final core data set.

## Results

Of the 17 invited experts, 16 expressed willingness to participate. Fifteen experts returned their proposals during stage one and two, and ten experts attended the consensus meeting. The final core data set was sent to all 15 experts.

In stage one, a total of 361 unique variables were proposed by the experts (additional file 1). After the aggregation of variables with similar content by the project group, 162 different variables were returned to the experts in stage 2 (additional file 2). During stage 2, 98 variables were rated and included in the preliminary data set discussed at the consensus meeting (additional file 3).

The final core data set consisted of 45 variables (Table 1,2,3,4 and 5) of which seven consisted of the same variable (physiological data) measured at two different time points (first and last values).

**Table 1:** Fixed system variables.

Data point number	Data point name	Field format	Data point categories or values	Type*	Exact definition of data point	Reference/Comments
1	Speciality of physician	Nominal (categorical data)	1 = Anaesthesiology	Bullet list	Specialist/non-specialist is described in "Description of system" section. Mixed refers to both anaesthesiology and emergency medicine	Proposed by project group
			2 = Emergency medicine			
			3 = Mixed			
			4 = Other			

2	Population	Continuous (numerical data)	Number		Annual number of citizens in area covered by service.	EED report (European Emergency Data Project)

3	Geographical service area provision		squared km		Area in which the service is planned to operate, squared km	EED-report

4	MD unit hours per year	Continuous	hh:mm		Unit hour is defined as the annual sum of hours the unit is occupied on a mission. ∑ time (patient arrival at hospital) - alarm time/year	EED-report

5	Mode of transportation accessible for the service	Nominal (categorical data)	1 = Ground	Check box	Mode of transportation vehicle(s) available to service on a regular basis.	Abbreviated from Utstein Trauma Registry 2008
			2 = Rotor-wing			
			3 = Fixed-wing			
			4 = Other			

6	Operating hours	Ordinal	1 = Full time service	Bullet list	Full time service is operational all days and nights	Proposed by project group
			2 = Day time service		Part time service is operational only day time and/or in hours of light	
			3 = Other			

7	Activation criteria	Nominal (categorical data)	1 = Criteria-based	Bullet list	Service is activated in accordance with a pre-defined set of activation criteria used by Emergency Medical Communication Centre	Proposed by expert panel
			2 = Consultation with physician		Service is activated only after consultation with an on-call physician	
			3 = Both			

8	Number of events per year	Continuous (numerical data)	Number		Events includes dispatches and requests in which the physician in service is involved when on call	"Responses/missions" recoded to "events" by project group

9	Dispatch system	Nominal	1 = Integrated Emergency Medical Communication Centre (EMCC)	Bullet list	Integrated EMCC includes dispatch centres coordinating all levels of pre-hospital services	Proposed by expert panel
			2 = Special EMCC		Special EMCC includes centres only responsible for physician-staffed pre-hospital units	
			3 = Both			

* "Bullet list" means only one possible answer. "Check box" means multiple answers possible.

hh:mm: hours: minutes

**Table 2:** Event operational descriptors.

**Data variable no**.	Data point name	Type of data	Data point categories or values	Type	Definition of data variable	Reference/comment
10a	Call received at Emergency Medical Communication Centre (EMCC)	Continuous	hh:mm		The time when the alarm call is answered at the initial EMCC	Utstein Trauma Registry 2008, Utstein Dispatch 2008

10b	Unit arrival on scene	Continuous	hh:mm		The time when the vehicle stops at a location as close as possible to the patient	Utstein cardiac arrest, 2004

10c	Patient leaving scene	Continuous	hh:mm		The time when the patient is transferred from the original location or time of death if dead on scene	ROC Epistry CA 2005, proposed revision from project organiser: change "from scene" to " from original location"

10d	Patient arrival at hospital		hh:mm		The time when the patient is formally transferred to receiving medical facility personnel	Proposed by project group

11	Type of dispatch	Nominal	1 = Primary medical mission	Bullet list	Includes all primary missions other than trauma (medical, surgical, paediatric and obstetric)	Proposed by expert panel
			2 = Primary trauma mission			
			3 = Interhospital transfer mission			
			4 = Search and rescue mission			
			5 = Consultation			
			6 = Other			

12	Type of transportation	Nominal	1 = Ground ambulance	Check box	Main type of vehicle used to transport the patient to definitive care.	Abbreviated definition according to Utstein Trauma Registry 2008
			2 = Helicopter ambulance			
			3 = Fixed-wing			
			4 = Other			
			5 = No transportation			

13	Result of dispatch	Nominal	1 = Patient attended	Bullet list	Dispatch means unit alarmed for mission or request/advice/supervision	Proposed by expert panel
			2 = Mission aborted due to weather			
			3 = Mission aborted due to technical reasons			
			4 = Mission aborted not required			
			5 = Mission aborted alternative tasking			
			6 = Supervision/advice only			

hh:mm: hours: minutes

**Table 3:** Patient descriptors

Data variable **No**.	Data variable name	Type of data	Data variable categories or values	Type	Definition of data variable	Comments
14	Age	Continuous	Number		The patient's age at the time of event	Utstein Trauma Registry

15	Gender	Nominal	1 = Female	Bullet list	The patient's gender	Utstein Trauma Registry
			2 = Male			
			3 = Unknown			

16	Co-morbidity	Ordinal	1 = No (ASA-PS = 1)	Bullet list	ASA-PS definition	American Society of Anaesthesiologists
			2 = Yes (ASA-PS = 2-6)		1 = A normal healthy patient	
			3 = Unknown		2 = A patient with mild systemic disease	
					3 = A patient with severe systemic disease	
					4 = A patient with severe systemic disease that is a constant threat to life	
					5 = A moribund patient who is not expected to survive without operation	
					6 = A declared brain-dead patient whose organs are being removed for donor purposes	

17	Medical problem (main reason for response)	Nominal	1 = Cardiac arrest	Bullet list	Select the condition most likely to be the patient's true medical problem	Proposed by project group. Defined according to EED-project and ScanDoc phase 1b. The first 9 categories should include >95% of all medical conditions present in daily work
			2 = Trauma			
			3 = Breathing difficulties			
			4 = Chest pain			
			5 = Stroke			
			6 = Acute neurology excluding stroke			
			7 = Psychiatry including intoxication			
			8 = Obstetrics and childbirth			
			9 = Infection			
			10 = Other			

18	Dominating type of injury	Nominal	1 = Blunt	Bullet list	Indication of the type of injury produced if trauma	Utstein Trauma Registry
			2 = Penetrating			
			3 = Unknown			

19 a)	Glasgow Coma Scale-first	Ordinal	Number (3-15)		First recorded pre-interventional Glasgow Coma Scale upon arrival of physician-staffed service	
b)	Glasgow Coma Scale- last		Number		Glasgow Coma Scale at end of care or patient hand-over	

20 a)	Heart rate first	Continuous	Number (per minute)		First heart rate per minute measured by physician-staffed service	
b)	Heart rate last		Number		Heart rate per minute at end-of-care or patient hand-over	

21 a)	Systolic blood pressure-first	Continuous	Number (mmHg)		First recorded systolic blood pressure measured by physician-staffed service (measured with sphygmomanometer, monitor or intra-arterial line).	
b)	Systolic blood pressure-last		Number		Systolic blood pressure at end-of-care or patient handover	

22 a)	Cardiac rhythm-first	Ordinal	1 = Sinus rhythm	Bullet list	First cardiac rhythm interpreted by physician-staffed service (minimum 3-channel lead)	
			2 = SVES, VES_mono_			
			3 = Atrial Fib/Flutt, AV-block gr. II/III, VES_poly_			
			4 = VF, VT, Asystole, PEA			
			5 = Not recorded			
b)	Cardiac rhythm-last		1 = Sinus rhythm		Cardiac rhythm at end of care or patient hand-over	
			2 = SVES, VES_mono_			
			3 = Atrial Fib/Flutt, AV-block gr. II/III, VES_poly_			
			4 = VF, VT, Asystole, PEA			
			5 = Not recorded			

23 a)	SpO2-first	Continuous	Number (0-100)		First recorded oxygen saturation by physician-staffed service (measured with pulse oxymeter or arterial blood gas (SaO2))	
b)	SpO2-last		Number		Oxygen saturation at end-of-care or patient hand-over	

24 a)	Pain-first	Ordinal	1 = None	Bullet list	First level of pain assessed by physician-staffed service	
			2 = Moderate			
			3 = Severe			
b)	Pain-last		1 = None		Level of pain at end of care or patient hand-over	
			2 = Moderate			
			3 = Severe			

25 a)	Respiratory rate-first	Continuous	Number (per minute)		First respiratory rate per minute measured by physician-staffed service. If mechanically ventilated, document ventilation rate.	
b)	Respiratory rate-last		Number		Respiratory rate at end of care or patient hand-over	

ASA-PS: American Society of Anesthesiologists Physical Status Classification System

SVES: supraventricular extrasystole, VES_mono_: single ventricular extrasystole, Atrial Fib/Flutt: atrial fibrillation or flutter, AV-Block: atrioventricular block, VES_poly_: polymorphic ventricular extrasystoles, VF: ventricular fibrillation, VT: ventricular tachycardia, PEA:pulseless electrical activity

**Table 4:** Process mapping.

Data **variable no**.	Data variable name	Type of data	Data variable categories or values	Type	Definition of data variable	Comments
26	Diagnostic procedures	Categorical	1 = US/Doppler	Check box		Proposed by expert panel
			2 = ECG- analysis (12-lead)			
			3 = Invasive monitoring			
			4 = Other point-of-care tests			
			5 = Point-of-care lab tests			
			6 = Other			

27	Therapeutic procedures					

27a	Drugs to facilitate airway procedure	Categorical	1 = Sedatives	Check box		Utstein Airway
			2 = Neuromuscular blocking agents			
			3 = Analgesics/opioids			
			4 = Local/topic anaesthetics			
			5 = None			

27b	Device used in successful airway management	Categorical	1 = Bag Mask Ventilation	Bullet list	Device used for successful airway management (device in place at end of care or patient hand-over)	Utstein Airway
			2 = SAD			
			3 = Oral TI			
			4 = Nasal TI			
			5 = Surgical airway			
			6 = None			
			7 = Unknown			

27c	Breathing- procedures used	Categorical	1 = Assisted manually	Check box	Open-airway manoeuvres or positioning of patient without use of any technical airway device (chin-lift, jaw thrust, recovery position)	
			2 = Assisted mechanically		Open-airway manoeuvres, including use of technical devices (guedel pattern, naso-pharyngeal airway)	
			3 = Controlled manually		Breathing assistance using physician's hands (bag-valve-mask ventilation)	
			4 = Controlled mechanically		Breathing assistance using technical respiratory support (ventilator, NIV)	
			5 = Chest tube/decompression			
			6 = Thoracostomi			
			7 = Other			
			8 = Unknown			

27d	Circulation- procedures used	Categorical	1 = Peripheral IV-line	Check box		
			2 = Central IV-line			
			3 = IO-Access			
			4 = Defibrillation			
			5 = Cardioversion			
			6 = Pacing			
			7 = Haemostatic, basic			
			8 = Haemostatic, advanced			
			9 = Other			
			10 = None			

27e	Disability- procedures used	Categorical	1 = Reduction of fractures	Check box		
			2 = Spinal immobilisation			
			3 = Therapeutic hypothermia			
			4 = Other			
			5 = None			

28	Medication, drugs administered		1 = Yes	Bullet list	Indicates whether medication was given by physician-staffed service. Exclude iv-fluid given for "keep-line-open" purposes	
			2 = No			

29	Type of medication	Categorical	1 = Analgesics/Opioids	Check box		
			2 = Sedatives			
			3 = Neuromuscular blocking agents			
			4 = Vasoactive			
			5 = Fibrinolytic			
			6 = Antibiotics			
			7 = Other			
			8 = None			

US: ultrasound

SAD: supraglottic airway device

TI: tracheal intubation

NIV: non invasive ventilation

IO-access: intraosseous access

**Table 5:** Quality indicators and mission outcome.

Data Variable **No**.	Data variable name	Type of data	Data variable categories or values	Type	Definition of data variable	Comments
30	Physiological improvement from SpO2, RR, HR, cardiac rhythm, SBP, GCS and pain				The expert panel recommends developing Quality Indicators based on changes in physiological parameters.	
31	Mission outcome	Nominal	1 = Left at scene	Check box		Proposed by expert panel
			2 = Patient to hospital, not escorted by physician			
			3 = Patient to hospital, escorted by physician			
			4 = Declared dead on arrival at hospital			
			5 = Declared dead at scene			

SpO2: oxygen saturation

RR: respiratory rate per minute

HR: heart rate per minute

SBP: systolic blood pressure

GCS: Glasgow coma scale

After stage 4, six experts returned minor comments related to a small number of the established definitions. A few minor changes were made accordingly. Moreover, some issues raised in this stage may be important for future revision of the data set. A revision of the template is planned in approximately two years.

The "fixed system variables" section contains nine variables, all of which relate to the context of care (Table 1). In the "event operational descriptors" section, seven variables were included derived from four time variables (Table 2). In the third section, "patient descriptors", nineteen variables were decided upon (Table 3). Eight variables were included to describe the diagnostic and therapeutic interventions within the episode of care in the "process mapping" section (Table 4). Finally, 2 variables were included in the "quality Indicator and mission outcome" section (Table 5).

## Discussion of core variables

### Background information and fixed system variables

The expert panel recommended that a description of the system under investigation be reported. These data will typically be "fixed", as they are not patient specific. In cooperative research, these variables can significantly influence comparisons between systems [40-42]. The expert panel divided this section into two parts; one related to the service's context (funding, integration within local EMS, medical capacities, training programs required) and the other including more specific variables, such as medical specialty required for staffing physician, population and service area covered, mode of transportation, operating hours and dispatch system. Strictly defining these variables makes it more straightforward to analyse the influence of system factors.

### Event operational descriptors

#### Mission times

Most physician-manned pre-hospital services document time variables, but there is no international consensus of the definition of these variables. Since emergency care is often time-critical, standardising how time is documented seems paramount. The expert panel agreed upon documenting time variables as exact times (hh:mm) and not as intervals. Most data registration tools will easily calculate the desired intervals, and it was thought that exact times would provide greater flexibility in statistical handling than intervals.

#### Call receipt at Emergency Medical Communication Centre (EMCC)

Response time is commonly documented, and frequently defined as the time from alarm to crew arrival at the scene. This time interval indicates the system's ability to respond to an emergency in a timely manner and is used as a quality indicator in the European Union [43]. The expert panel realised that the receipt of the emergency call at the EMCC is more difficult to collect than the actual alarm time to the attending crew. Nevertheless, the time of receipt of the emergency call from the scene is closer to the actual out-of-hospital time from the patient's perspective, and the expert panel agreed to include this time as the "start of event" indicator.

#### Type of dispatch

This indicator variable is important for benchmarking purposes [44], and will not only describe the crude mission profile of the service, but will also be useful for the stratification of mission type data extraction. Some physician-staffed pre-hospital services are dispatched to undifferentiated emergency calls but many are targeted to particular types or severity of incident [6,7]. A substantial workload is imposed onto some services, because of consultation responsibilities for lower-level services (paramedics, dispatch centre) [6]. The expert panel emphasised that this function should also be documented.

### Patient descriptors

#### Co-morbidity

Robust risk-adjustment measures are fundamental for any effort to compare the characteristics of care against hard outcomes [45]. Clearly, the same treatment for the same medical problem can yield different outcomes in groups of patients with different levels of co-morbidity [46]. The expert panel included the well-established and recommended American Society of Anesthesiologists Physical Status Classification System (ASA-PS) [47], albeit in a dichotomised form, as ASA-PS = 1 or ASA-PS>1, as an indicator of co-morbidity. It was considered that while it would be ideal to use the full ASA-scale, obtaining the complete medical history from seriously ill patients is likely to be impossible. It is emphasised that the patient's morbidity prior to the acute medical incident is to be recorded.

#### Medical problem

It is crucial for benchmarking to have key variables to serve as inclusion criteria. Because an accurate diagnosis is difficult to establish in the pre-hospital setting [48], the expert panel recommended inclusion of a complaint-based indicator for the medical reason for dispatch to better reflect the real-world setting.

#### First and last values

As monitoring technology evolves, the amount of vital patient data is almost unlimited, and monitoring strategy is often case-specific. The expert panel recommended documenting these vital data: Glasgow Coma Scale [49], heart rate, systolic blood pressure, cardiac rhythm, oxygen saturation (SpO2) and respiratory rate. Typically, such measurements are recorded in a continuous pattern but reporting a complete dynamic profile of the patient's physiology during the process of care will be impractical. The expert panel recommended to report the first and last of these measurements. This approach will form the basis for later analyses of therapeutic and diagnostic interventions related to the patient's clinical condition within the episode of care [50].

#### Cardiac rhythm

Advanced anti-arrhythmic treatment is usually confined to physician-staffed pre-hospital services. Several experts noted that recording the cardiac rhythm is often unreliable and not always indicated. Nevertheless, interpretation of cardiac rhythm and treatment of malignant arrhythmias is clearly an advanced intervention. This intervention should be documented, and a four-point scale was accordingly proposed. This scale is inspired from the Mainz Emergency Evaluation Score and separates the cardiac rhythm into normal (sinus rhythm), pathological (atrial fibrillation, AV-block grade 1-2), pathological with circulatory compromise, and cardiac arrest [51]. The expert panel believes that this classification can be applicable using a 3-channel electrocardiogram monitor.

#### Pain

Adequate analgesia is regarded as good clinical practice [52], is one major task for pre-hospital services, and should be documented accordingly [53]. There is currently no consensus on which method for documenting pain is most applicable in a pre-hospital setting [54]. Several of the experts argued that the visual analogue scale (VAS) should be used because it is the most established scale in pain assessment. Nevertheless, use of the VAS requires a conscious and cooperative patient, which is not always available in emergency care. A reasonable approach is to document a more coarse scale assessed by the physician. The experts recommend including a three-level pain scale was chosen, consisting of the following classifications: no pain, moderate pain and severe pain.

### Process mapping

Diagnostic procedures were included in an effort to document the specially trained physician's competency in diagnostics. The expert panel agreed that this role in diagnostics will probably be more important in the future.

Therapeutic procedures were systemised according to the traditional ABCD algorithm, and the procedures used in treatment of each were recommended for reporting accordingly.

#### Medications

The expert panel agreed upon dividing the medication variable into two types. The first type describes whether any medication was administered, whereas the second documents which type of medication was administered. Emphasis is placed on the type of medication that usually is administered only by physicians. These medications include potent analgesics and sedatives, neuromuscular blocking agents, vasopressors, intravenous antibiotics and anti-arrhythmics.

### Quality indicator and outcome measure

The expert panel suggested the outcome measure should be a description of mission outcome rather than a specific patient medical outcome. Although the recording of long-term patient outcome is ideal it was recognised that this is not achievable in many systems because services often transport patients to different locations and medical facilities.

The expert panel did not agree upon a universal quality indicator during the consensus process. Nevertheless, there seems to be a common opinion that such quality indicators should be developed, and that improvement or deterioration in the clinical condition during the episode of care can serve as a relevant and patient-centred quality indicator. How such a quality indicator should be developed was outside the scope of this consensus process. Several proposed variables can prove useful for selection criteria in future benchmarking and research: variable 11, "type of dispatch", will indicate the main operational indication for dispatch, whereas variable 17, "medical problem", will further indicate the medical basis for dispatch. When establishing categories within these variables, a compromise between precision and user-friendliness must be achieved; too many categories will be accompanied by difficulties in definitions, whereas too few will result in loss of precision. Therefore, an extended version of the "first-hour quintet" is proposed which [43] will include > 90% of the medical complaints met in daily service for most services (unpublished data from ScanDoc phase 1b).

## General discussion

### Primary findings

Using experts within the field and the modified nominal group technique, we defined a core data set to standardise documenting and reporting in physician-staffed pre-hospital care.

If implemented, we believe that shared research efforts within the field will be enhanced.

The fairly strict definitions and categories within each variable will form the basis for robust documentation and facilitate the extraction of comparable data. The established variables are recommended to reflect ordinary pre-hospital care but also seek to document variables on the specific functions of specially-trained physicians and their exclusive diagnostic and interventional capacities. In addition to being experts in securing vital functions (airway, breathing and circulation), these physicians can apply more aggressive pain control, interpret electrocardiograms and perform advanced diagnostics [8,55-58]. The expert panel agreed on the importance of robust documentation of these advanced interventions, especially those related to the ongoing debate on whether advanced pre-hospital care improves patient outcome.

Shared research efforts require unique patient group identifiers. In the current core data set, several variables can act as such identifiers. Traditionally, pre-hospital treatment strategies have been symptom-based. A diagnostic uncertainty is generally present, and interventions are applied according to the physician's preliminary diagnosis. In the dispatch phase, this diagnostic uncertainty is even more present. Nevertheless, as more advanced diagnostic equipment, such as ultrasound and near patient biochemical tests, are commonly available, diagnostic precision may improve. However since this level of diagnostic equipment is not always accessible, a focus on chief complaints remains fundamental.

### Implementation of core data

The expert panel emphasised that it should be possible to use the core data set without replacing the current infrastructure of documentation. This project does not intend to construct a unique documentation software, as this software could interfere with already developed and well-functioning local systems. Therefore, we believe the main benefit of this data set is to standardise current reporting and create increased understanding of how variables should be documented and defined in the future.

### Limitations

Using a consensus methodology to develop a core data set for physician-manned pre-hospital services presents methodological challenges exist [38]. First, there is a risk of selection bias when appointing experts. We believe that the multi-national selection of experts with predefined selection criteria reduced the risk of establishing a biased expert panel. Second, a well-known risk related to the project group's influence on the expert panel is a provision of biased literature before the first round [31]. The project group did provide the expert panel members with a selection of background literature because previously developed and implemented templates were too important to be neglected. Nevertheless, the possibility of this "literature-based" bias seems minimal, given the huge number of variables proposed in the first round.

The specific operational and medical characteristics of the EMS connected to individual experts might have influenced the priorities and proposals during the process. This problem was evident during the consensus meeting, but the panel agreed that the proposed core data set was feasible for most mission types. It is underlined that the current data set represents a minimum, and supplementary variables usually are documented according to service preference.

## Summary and conclusions

Using a modified nominal group technique, we have established an Utstein-like template for documenting and reporting in physician-staffed pre-hospital services. The core data set consists of 45 variables grouped in five different categories. We recommend that future studies in the field report their data according to the template. We believe the template can facilitate future shared research efforts in advanced pre-hospital care.

## Competing interests

The authors declare that they have no competing interests.

## Authors' contributions

AK and HML conceived and organized the study. AK, HML organised, and DL, HML and SDB moderated the consensus meeting. AK and HML drafted the manuscript, and all authors read and approved the final manuscript.

## Supplementary Material

Additional file 1 **Results Stage 1**. All proposals from experts after completion of stage 1 Click here for file

Additional file 2 **Results Stage 2**. All rankings from experts on proposals from stage 1 Click here for file

Additional file 3 **Proposals before consensus meeting**. All proposals organized by the sum of ranking points from stage 2. Click here for file
