# Supplementary material for: A consensus-based template for documenting and reporting in physician-staffed pre-hospital services
Source: Scand J Trauma Resusc Emerg Med. 2011 Nov 23;19:71. doi: 10.1186/1757-7241-19-71 (PMC3282653; doi:10.1186/1757-7241-19-71)
Supplement: Additional file 1 — Results Stage 1. All proposals from experts after completion of stage 1 [file 1757-7241-19-71-S1.PDF]

The Utstein Template for documenting and reporting in physician-staffed pre-hospital se

AIM: To establish a common core data set with definitions for activity documentation and shared research efforts. Core variables s

Proposals, first round (For an explanation of the terms and methodology please see the attached paper (Ringdal, Coats
Structure your proposals for five core and up to five optional variables according to the six categories listed below. If the variable is c
Each variable should be accompanied by an exact definition and from where the variable can be extracted (source of information), if

We strongly encourage use of the attached templates and datasets for definition purposes.

Data variables with exact definitions:

Fixed system variables.

Definition: Variables crucial for comparisons between services and/or countries. Ask yourself: "what would I like to kno

Data point nb	Data point	Core/optional	Variable categories	Source of information
1	Type of transportation	core		Type of transportation delive
2	Highest level of patient care			
3	prehospital care at all			
4	prehospital airway mangement			
5	occupation/experience from provider/assistant			
6	trauma/internal/mix of missions, OB-GYN, newborn			
7	Number of missions/pat pr yr			
8	secondary missions-interhospital transfer, incubator			
9	24 hrs service/not			
10	state/commercial/private funded			
1	Educational level	Core	Predfined string: Type of	When enrolling a service to
2	Training level	Core	Number: Number of year	Physician
3	In-hospital training	Core	Number: Number of mon	Physician
4	In-hospital training in what type of	Optional	Pedfined string: Type of c	Physician
5	Assistant	Core	Predfined string: When p	Physician
6	Opening hours	Core	Predefined string: When i	Physician

7	Equipment	Core	Predfined string: types of equipment	
1	**inhab/km2**	core		
2	median response time ambulance	core		
3	tier response	core		
1	crew composition	core	1 physician+1nurse 2 physician+2 nurses 3 physician	
2	MD-ALS unit hours per 100,000	core		
3	MD-ALS unit hours (service area)	core		
4	seniority and training of physician e	core	free text	
5	Non MD-ALS unit hours per 100,00	core		
6	Non MD-ALS unit hours (service ar	optional		
1	Population	Core	Number	
2	Area	Core	Number	
3	Mission types	Core		
4	Vehicles available	Core		
5	Crew composition	Core		
6	Type of hospitals in coverage area	Optional		
1	**inhab/km2**	core		
2	median response time ambulance	core		
3	tier response	core		
1	Level of proficiency	Core	1 = Resident; 2 = Reg. s	Responding unit
2	Medical branch	Core	Ordinal	Responding unit
3	Number of medical personnel	Core	Continuous	Responding unit
4	Level of assisting personnel	Core	Ordinal	Responding unit
5	Mode of transportation	Core	Nominal	Responding unit
6	Categorization of missions	Optional	1 = Primary; 2 = Inter-h	Responding unit
7	Activation criteria	Optional	1 = criteria based; 2 = c	Responding unit
8	Nb of inhabitants	Optional	Continuous	Responding unit
9	Annual nb of responses	Optional	Continuous	Responding unit
1	Size of population served per unit	Core		
2	Response time	Core		
3	Percentage of physician assisted ru	Optional		
4	Area served per unitCore			
5	Percentage of runs aborted en rout	Optional		
1	distance/time to trauma center			

2	Intubation rate	Core		
3	blunt/penetrating	Core		
4	type of prehospital care	Core		
5	M,W (s), Z statistic	Core		
6	ISS	Core		
1	experience of physician in HEMS	optional	yr	from system involved
2	fixed base		yes/no	from system involved
3	team composition	core	paramedic/nurse/HCM-pa	from system involved
4	dispatch system	core	alarm center/special HEM	from system involved
5	physician backroundcore		anesth/int med/trauma/	from system involved
6	mode of trasport the team	core	helicopter/ambulance/rap	from system involved
1	Highest Level of EMS provider on scene	core	1 = EMS non-P<br>2 = EMS-P<br>3 = nurse<br>4 = physician non Anaesthesia trained<br>5 = Anaesthesia trained Emergency Physician	Highest level of EMS provider on scene, excluding any non-EMS personell (bystanders, family etc)
2	Rescue system	core	1 = fixed system, 2 = Rendez-vous system	
3	mode of transportation	core	1 = ground ambulance<br>2 = helicopter ambulance<br>3 = fixed-wing ambulance<br>4 = not transported<br>5 = unknown	Main type of transportation vehicle (if multiple chose vehicle used for the majority of the transportation phase)
1	Population / socio-demograhic data	core	scoring according to inhabitants / km2	
2	Geographic data	core	location of the emergency (i.e. public place, road, higl	
3	qualification of physicians	core	(i.e. anesthesiologist, ems physician...)	

Event operational descriptors

Definition: Variables related to indication for dispatch, timelines for event and logistics.

Data point nb	Data point	Core / optional	Variable categories	Source of information
1	emergency, mandatory,			
2	time of day			
3	time of year			
4	type of transportation			
5	dispatch type, surgical, trauma, medical			
6	Time from alarm to arrival at scene			
1	Type of dispatch	Core	Predefined string:	Physician
2	Mission completion	Core	Predefined string:	Physician
3	Reason for aborted mission	Core	Predefined string:	Physician
4	Response time	Core	Number (minutes):	Physician
5	Driving time	Core	Number (minutes):	Physician
6	On scene time	Core	Number (minutes):	Physician
7	Transport time	Core	Number (minutes):	Physician
8	Delayed	Core	Yes/No:	Physician
9	Specific reasons for prolonged on s	Core	Predfined string:	Physician
10	Other resource on site +/- 5 minut	Core	Predfined string:	Physician
1	alarm time			
2	dispatch time			
3	**dispatch code (level of acuity)**			
4	arrival on scene			
5	departure from scene			
6	arrival hospital			
1	type of event	core	1medical 2 injury 3 unknown	
2	dispatch code	core	1a highest priority, cardiac arrest 1b highest priority 2	
3	verified code	optional	as above	
4	Time from alarm to arrival at scene	core		
5	Time from alarm to hospital arrival	optional		
6	Means of transport to scene	optional	1 ground ambulance 2 car 3 helicopter 4 other	
7	Scene time	core		
8	Patients treated by physician	optional	1 one 2 two 3 > two	
9	Total number of units dispatched	optional		
10	Date and time of call	core		
1	Mission/task type	Core		

2	Activation time (Alarm-vehicle mov	Core		
3	Response time (Vehicle moves-On s	Core		
4	Mission not completed	Core		
5	Highest level of prehospital care pr	Optional		
1	alarm time			
2	dispatch time			
3	**dispatch code (level of acuity)**			
4	arrival on scene			
5	departure from scene			
6	arrival hospital			
1	Initial call receipt	Core	Hours and minutes	Initial public safety answerin
2	Activation of unit	Core	Hours and minutes	Responding unit or dispatch
3	Unit mobile	Optional	Hours and minutes	Responding unit or dispatch
4	Arrival on scene	Core	Hours and minutes	Responding unit or dispatch
5	Departure from scene	Core	Hours and minutes	Responding unit or dispatch
6	Arrival at receiving facility	Core	Hours and minutes	Responding unit or dispatch
7	Reason for dispatch	Optional	Nominal	Dispatch criteria
8	Type of transportation	Optional	Nominal	Responding unit
1	Alarm time	Core		
2	Dispatch time	Core		
3	Arrival at the scene	Optional		
4	Departure from scene	Optional		
5	Arrival at hospital	Optional		
6	Total time in service	Core		
1	High energy trauma	Core	by mechanism of trauma or by physiological paramete	
2	logistical reasons	Core		Distance or road traffic
3	Prehospital time	Core		
4	On scene times	Core		
1	dispacth time	core	min	case file
2	time to patient	core	min	case file
3	time at scene	core	min	case file
4	transport time	core	min	case file
5	way of transport	core	ambulance doc escort/am	case file
6	cancelled	core	no go/ no weather/on-rou	case file

7	cancelled from scene	core	no need/	case file
8				
9	time of mission	core	time of day	case file
1	Trauma	core	1 = traffic, 2 = occupational, 3 = leisure/sports, 4 = c	
2	Medical	core	1 = ACS, 2 = stroke, 3 = other cardiovascular, 4 = ai	
3	Paediatric	core	1 = airway/breathing, 2 = cardiovasc., 3 = seizure, 4	
4	Obstetric/gynecol.	core	1 = bleeding, 2 = eclampsia, 3 = pregnancy other	
5	mode of mission		1 = primary, 2= transfer	
6	type of destination hospital	core	1-3 = Level 1-3	
1	time logistics (utstein template dis	core	times	
2	type of response	core	(i.e. ambulatory care, transport without physicians, t	
3	scoring of target hospital	core	i.e. comprehensive care, advanced care, basic care)	
4	dispatch diagnosis	core		
7				
8				
9				
10				

Patient descriptors

Definition: Patient characteristics such as age, gender, co-morbidity eg.

Data point nb	Data point	Core / optional	Variable categories	Source of information
1	rts delta/MESS			
2	age			
3	gender			
4	ASA-PS			
5	co-morbidityty			
6	prehospital airway management			
7	RR			
8	GCS			
9	Vital data before and after treatment			
10	SpO2			
1	Gender	Core	M/F	Physician
2	Age	Core	Number (years)	Physician
3	Co-morbidity	Core	String (predfined):	Physician

4	Drug-abuse	Core	String (predfined):	Physician
5	In trauma: position in vehicle	Core	String (predfined):	Physician
6	In medical: situation of patient	Core	String (predfined):	Physician
7	Medical problem (main reason for r	Core	ICD-10	Physician
8	Surgical problem (main reason for	Core	ICD-10	Physician
9	GCS	Core		Physician
10	RTS	Core		Physician
2	pain assesment	core		
1	Age	core		
2	gender	core		
3	comorbidity	core		
4	key diagnosis if medical	core		
5	mechanism of injury if injury	core	as from Utstein Trauma Registry + burns and drownin	
6	ISS if injury	core		
7	cardiac arrest	core	1 yes on arrival 2 yes after arrival 3 never	
8	AIS region(s) with score>2	optional	AIS regions	
9	GCS on arrival	optional		
10	SBP on arrival	optional		
1	Age	Core		
2	Gender	Core		
3	ASA-PS	Core		
4	Patient Category	Core		
5	Dominating type of injury	Optional		
6	Dominationg type of medical incide	Optional		
7	RTS			
1	Age	Core	Continuous	Responding unit or dispatch
2	Gender	Core	1 = Female; 2 = Male; 3	Responding unit or dispatch
3	Co-morbidity	Core	Ordinal (1-7) according t	Responding unit or main tre
4	Severity	Core	Ordinal (1-7) according t	Responding unit
5	Tentative diagnosis	Core	Ordinal	Responding unit
6	External causes	Core	Ordinal	Responding unit
7	Initial GCS	Core	Eye-, verbal- and motor-s	Responding unit
8	GCS on admission	Optional	Eye-, verbal- and motor-s	Responding unit
9	Initial RTS	Core	Ordinal (according to Uts	Responding unit

10	RTS on admission	Optional	Ordinal (according to Uts	Responding unit
1	Age	Core		
2	Gender	Core		
3	Alarm code	Core		
4	Preexisting ASA classification	Core		
5	Respiratory Rate	Optional		
6	Oxygen saturation	Optional		
7	Blood pressure (Sys and Diastol)	Optional		
8	GCS			
9	Survival status upon leaving patient			
1	Co-morbidity	Core		
2	age	Core		
3	gender	Core		
1	sex	core	male/female	case file
2	age	core	>1 yr, 1-5 yr, 5-15 yr, 1	case file
3	co-morbidity	core	ASA I-V	case file
4	reason for alarm		cardiac/seizure/trauma/..	case file
5	condition when met compared to al	core	same/worse/better	case file
6	GCS	core	3-15	case file
7	BP(systolic)	core		case file
8	HR	core		case file
9	rhytm	core	SR/FA/SVT/etc	case file
10	ICD 10 diagnose (rough)	core		case file
11	RTS (trauma pat)		0-12	case file
1	age	core		
2	gender	core		
3	severity	optional	NACA-Index	
4	GCS-category	core		
5	RTS	optional		
6	BP categories	optional	1 = >90, 2 = <90	
7	HR categories	optional	1 = <100, 2 = >100	
1	social situation	core	(i.e. homeless, deprivation, criminal background...)	
2	basic data	core	age, gender...	

Process mapping

Definition: Variables related to what happened to the patient, such as treatments and procedures performed.

Data point nb	Data point	Core/optional	Variable categories	Source of information
1	Time from alarm to arrival at scene			
2	Key intervention			
3	Airway management			
4	Drainage			
5	Sedated/medication			
6	Immobilised			
7	Hemostasis			
8	Ventilator			
9	Incubator			
10	CPR			
1	Other persons at site?	Core	Y/N	Physician
2	Basic medical help provided by bys	Core	Y/N	Physician
3	In CA: CPR started by bystander?	Core	Y/N	Physician
4	IN CA: Airway secured by other EM	Core	Y/N	Physician
5	If yes: type of airway management	Optional	String (predfined):	Physician
6	Diagnostic importance of physician'	Core	Y/N	Physician
7	Consequence: changed admittance	Core	Y/N	Physician
8	Theraperutic importance of phsysic	Core	Y/N	Physician
9	If yes: could treatment have been	Core	Y/N	Physician
10	What type of treatment was provid	Core	String (predfined):	Physician
1	securing the airway			
2	cricotomy			
3	trombolysis?			
4	cooling?			
1	intubation			
2	iv access			
3	intraosseus			
4	ultra sound exam			
5	blood test on scene			
6	pleural drainage			
1	procedures	core	1 IV line 2a IV drugs 2b fibrinolysis 3 nebulization 4 b	

2	immediate outcome	core	1 dead on scene-no treatment 2 dead on scene after t	
3	late outcome	core	1 alive 2 dead	
4	means of trasportation to hospital	core	1 ground ambulance 2 helicopter 3 other	
5	bystander CPR	core	1 yes 2 no	
1	Airway management	Core		
2	Vascular access	Core		
3	Need for breathing support	Core		
4	Need for circulatory support	Core		
5	Type of transport	Core		
6	Type of admitting health facility	Optional		
1	intubation			
2	iv access			
3	intraosseus			
4	ultra sound exam			
5	blood test on scene			
6	pleural drainage			
1	Airway intervention	Core	Nominal	Responding unit
2	Vascular intervention	Core	Nominal	Responding unit
3	Medication	Core	Nominal	Responding unit
4	Surgical intervention	Core	Nominal	Responding unit
5	Diagnostic intervention	Core	Nominal	Responding unit
6	Other intervention	Core	Nominal	Responding unit
7	CPR	Core	Nominal	Responding unit
1	Tracheal intubation			
2	Supraglottic airway device			
3	Positive pressure ventilation			
4	i.v. access			
5	i.o. access			
6	Ultrasound diagnostics			
7	Blood sample on scene			
8	Thoracic drainage			
9	Enrollment in scientific protocol			
1	ISS	Core		
2	intubation	Core		

3	GCS	Core		
4	RTS	Core		
1	airway	core	open/LMA(LT)/ET/trach	case file
2	ventilation	core	spont//CPAP/hand assist/	case file
3	pleural decompression	core	no/needle/open/drainage	case file
4	ECG (13/12-lead)	core	yes/no	case file
5	infusion	core	colloid/cristalloid/blood p	case file
6	medications	core	analg/cardiac/sedation/in	case file
7	defibrillation	core	yes/no	case file
8	cardioversion	core	yes/no	case file
1	Response time	core	Number	
2	Scene time	core	Number	
3	Transport time	core	Number	
4	Non-invasive interventions	core	1 = iv access, 2 = io access, 3 = iv/io drug, 4 = oxyg	
5	Invasive interventions	core	1 = et intubation, 2 = cricothyrotomy, 3 = chest tube	
6	Monitoring	core	1 = BP, 2 = pulse oximetry, 3 = ECG, 4 = capnograph	
7	Resuscitation	core	1 = chest compression, 2 = defibrillation	
8	Ventilation	optional	1 = manual BV, 2 = ventilator	
9	Adjuncts	optional	1 = telemetric ECG-transmission	
1	vital sings and monitoring paramet	core	to be defined, overlap from other utstein templates	
2	pain	core	VAS	
3	trauma mechanism	core	(i.e. blunt, penetrating)	
4	trauma history	core	(i.e. fall, car accident, burn...)	

Outcome measures or Quality Indicators- Optional

Definition: Suggest any outcome measures or quality indicators during the pre-hospital phase of care.

Data point nb	Data point	Core/optional	Variable categories	Source of information
1	Glasgow Coma Scale (GCS)			
2	upon arrival of EMS personnel at scene			
3	rts delta/MESS			
1	GCS at arrival and at arrival i hospital			
2	RTS at arrival and at arrival in hospital			
3	VAS (visual analogue pain score) at arrival and at arrival in hospital			
4	BP (MAP) at arrival and at arrival in hospital			

5	HR, RF at arrival and at arrival in hospital			
1	number of procedures before succes (above)			
1	dispatch code=verified code		1 yes 2 no, verified higher 3 no, verified lower	
2	first unit dispatched= highest level unit on scene		1 yes 2 no	
3	hospital of arrival= hospital of definitive treatment		1 yes 2 no	
1	Discharge destination	Optional	1 = Home; 2 = Rehab; 3	Responding unit or primary
2	Glasgow Outcome Scale	Optional	5 = Good Recovery; 4 =	Main treating hospital
3	Survival status	Optional	1 = Dead; 2 = Alive; 3 =	National registry
4	Final diagnosis	Optional	Ordinal	Main treating hospital
5	Abbreviated Injury Scale (AIS)	Optional	Ordinal	Main treating hospital
1	Any intended procedure not carried out			
1	W statistic	Core		
2				
3				
4				
5	30 day mortality	Core		
1	HEMS benefit score	core	0-8	case file
2	change in vital signs	core		case file
3	RTS on arrival to hosp	optional	0-12	case file
4	need for transport	core	yes/no	case file
1	ICU-Time	optional	Number	hospital
2	LOS in-hospital	optional	Number	hospital
3	mortality	Core	1 = dead on surrival, 2 = death on scene, 3 = death c	
4	GOS	optional		
1	GCS	core	worst before intervention vs. best after intervention	
2	NACA	core	worst before intervention vs. best after intervention	
3	vital parameters	core	worst before intervention vs. best after intervention	
4	MEES	optional	before and after intervnetion	
4				
5				

Individual expert member proposal- Optional

Definition: Variables suggested by expert panel member regarded to be important and to be included in round 2.

Data point nb	Data point	Core / optional	Variable categories	Source of information
1	preliminary diagnosis, medical diagnosis AMI, SAH,Respiratory disease (COPD, Asthma),circulatory disease,se			
2	head injuries like TIA, infarction/bleedings			
3	birth, newborn, children under 1, older children			
4	gynecology related, bleedings, inf,burns, drownings			
5	EtCo2, SpO2, RR, HR, SBP before and after treatment/management			
1	Complication	Optional	TBD	Responding unit
2	Hospital response	Optional	TBD	Responding unit
3	Valid alternatives	Core	TBD	Responding unit or dispatch
4	Validy of activation	Core	TBD	Reponding unit
1	Adherence to treatment protocols i	Optional		
1	Quality of life	optional	EQ-5D (and HUI)	Guidelines for the conductio
1	consultation			case file
2	from who	EMS/basic health care		case file
3	consultation resulted in			

ervices.

hould be possible to collect routinely, and should be easy to adapt to most excisting softwares.

et al).

categorical, please list the suggested categories.

available.

ow if I were to compare my results with another service?"

Exact definition of data point	Comments for discussion
ering the patient	
Specialist in anaesthesiology, in training for specialty in anaesthesiology, Specialist in emergency medicine, in training for speciality	
Months (full time work)	
% of full time work	important to register to what degree the physicians maintain procedures with in-hospital training
General anesthesia, Nevroanaesthesia, Heart, Gastrosurgical anaesthesia, Obsteric anaesthesia, Child anaesthesia, Intensive care wa	
HEMS Paramedic, HEMS anestheti	It is improtant to control all variables. The assistant is important - especially in unanticipated diffi
24/7, all week day and evening, all week only daytime, working days day and night, working days 24h, working days only daytime, (	

Ventilator (advanced), ventilator (simple), Defibrillator, Invasive BP-measurment tool, 12-lead ECG, 3 or 5 lead ECG, NO-inhalation	
	we need to know if we are operating in rural or city area
	To get an impression on the system
	is the doc car called out at once or later ?
the usual composittion of the physician-manned crew attending the patient on scene	
as from Unit hours ALS 'European Emergency Data project': Annual unit hours of ALS (physician only in this case) per 100,000 inhab	
Annual unit hours of physician ALS per km2 of service area	
same as in data point	
as above	
as above	
	we need to know if we are operating in rural or city area
	To get an impression on the system
	is the doc car called out at once or later ?
Attending physicians level of educ	Number of levels
Main medical branch of specialilty	Number an description of specialities
Number of crew-members with medical duties participating in response unit	
Level of assisting personnels medi	Definition of levels
Types of vehicels for scene-respon	Description of different types
Categorization of different types o	Description of different types
Description of how decision of response is taken	
Number of inhabitants in units are	Number or density? Migration?
Number of activated responses an	Definition

level of care/training	
1 = Physician and Paramedics use the same vehicle for patient approach	
hway)	

Exact definition of data point	Comments for discussion
Emergency medical mission, emergency trauma mission, Transfer of ICU patient from lower to higher level of treatment, transfer of	
Completed mission, Aborted mission, Mission handed over to other unit	
Weather, other higher priority mission, updated infor of no need for responce	
Time from alarm to initiation om mission	
The net driving (flying) tim to patient site	
The net time from reashinbg patient to start of transport	
The net driving (flying) time to hospital	
Yes: reason, No: reason	
Lack of resources, entrapped patient, difficulty evacuating patient from site to waiting ambulance, threats, need for assistance from	
Police, Firedep, Security, Social care, Home nurse, GP, MD passing by, Other health care proffessional passing by	
classification of event at the time of dispatch of first unit (1st unit may also have been non-physician)	
priority assigned at dispatch	
priority as judged by physician on	alternatively, the NACA score. It can also be used to cross-check the accuracy of dispatch
as from Utstein trauma registry	
as from Utstein trauma registry	
The type of vehicle that transported the physician to the place of event	
the number of rescue units dispatched to the event, including the one transporting the physician	
	Consultation/Primary medical/Secondary medical/ Rescue

	See trauma utstein
Time of earliest call received from	Definition of IPSAP
Time when crew of responding unit was notified	
Time when unit is mobile	
Time when responding unit arrives	Definition of "on scene"
Time when patient was transporte	One or two variables
Time when unit arrives at emergency department or hospital and/or transfer of treatment responsibility	
Categorization of main reason for	Which source or set of criteria?
Main type of transportation vehicle (if multiple; type used for majority of trsp phase)	
	Core variable if pertinent
	Core variable if pertinent
	Core variable if pertinent
In the event of ambulance run aborted en route	
Validity of helicopter emergency medical services dispatch criteria for traumatic injuries: a systematic review.	
Ringburg AN, de Ronde G, Thomas SH, van Lieshout EM, Patka P, Schipper IB.	
Prehosp Emerg Care. 2009 Jan-Mar;13(1):28-36. Review.	

other	
rway & breathing, 5 = seizure, 6 = unconsciousness, 7 = endocrinology, 8 = other	
= infectious, 5 = other	
ransport with physician, air lift, dead on scene)	

**Exact definition of data point**	**Comments for discussion**
Heart, Lung, Diabetes, GI, Neurological, Epilepsy, etc: Use the standard list from a anesthetic chart?	

Herione, Other opioid, Cocaine, Amphetamine, Other sentral stimulating drug, Cannabis, Benzodiazepines, Ither (define).	
Driver, passenger front, backseast right, backseast left, backseast middle	
Home, stairs, elevator, pavement, other outdoor (define)	
X.XX	
X.XX	
VAS score	
ASA classification as from Utstein trauma registry	
ICD9 or ICD 10 code of 1st diagnosis on hospital charts (EMS charts if patient left on scene)	
ng	
whether CA occurred at any time before arrival at hospital	
	See Utsein Airway
	See trauma utstein
The patient's age at the time of event	
The patient's gender	
ASA-PS Classification System (categorisation of pre-event comorbidity)	
Classification of the medical sever	Decision of scale and levels
Diagnosis based on prehospital evaluation according to ICD-10 (Ch I-IX)	
External causes of morbidity and mortality according to ICD-10 (Ch X)	
Initial GCS without interventions/qualifiers (incl. preverbal/pediatric)	
GCS on admission and/or after ne	Description of sedated/intubated pts
Revised Trauma Score (RTS) categories with clinical notes on scene (pre-intervention)	

Revised Trauma Score (RTS) categ	Description of sedated/intubated pts
Medical / Surgical / Trauma / Psychiatry	
If pertinent (Excluding missions without any patient contact)	
Following ASA-classification or by specific co-morbidities	
at first patient contact, and at handover hospital	
at first patient contact, and at handover hospital	

**Exact definition of data point**	**Comments for discussion**
(List of intervensions, f.eks fra Rapportskjema Statens Luftambulanse)	
	all these should ONLY include procedures made by the doctors
ag mask ventilation 5 supraglottic device 6 intubation & ventilation 7 chest drain 8 electric stimulation of the heart (includes defibrill	

treatment 3 transported alive to hospital	
outcome at 30 days	
whether a CA patient received at least cardiac massage before pysician arrival	
	See Utstein Airway
o2, CPAP,BiPAP,Pleural drainage,Ventilator	
Fluids, drugs	
Home/Health Care Centre/Primary Hospital/Secondary hospital/Tertiary hospital	
	all these should ONLY include procedures made by the doctors
Variables according to Utstein tem	Decision on nb of interventions
1 = volume replacement (TBD); 2	Decision on nb of interventions
According to ATC (4th level)	Decision on level of registration
1 = thoracostomy (incl needle-dec	Decision on nb of interventions
1 = Ultrasound; 2 = Invasive pres	Decision on nb of interventions
1 = CPR; 2 = defibrillation; 3 = ca	Decision on nb of interventions
Registration according to Utstein template for cardiac arrest and CPR	

separate socring og Eye,motor, verbal, since motor-score has most predictive value	
emergency call center to arrival at the patient	
arrival at the patient to start of transportation	
departure from scene to end of hospital handover	
en spont. breathing, 5 = BVMV, 6 = supraglottic device	
ny	

**Exact definition of data point**	**Comments for discussion**

The patient's destination after end of acute care in the initial (main) hospital	
Glasgow Outcome Scale – at discharge from main hospital	
Alive or dead 30 days after event	
Diagnosis on discharge according to ICD-10 (compared with tentative diagnosis)	
AIS severity codes that reflect the injuries	
	I.E: Intended intubation not possible.
J Trauma. 2005 Jun;58(6):1272-6; discussion 1277.	
M-study; arguments for regional trauma databases.	
Joosse P, Goslings JC, Luitse JS, Ponsen KJ.	
Trauma Audit Research Network (TARN)	
days	
days	
on transport, 4 = death in hospital, 5 = hospital demission	
Glasgow outcome score	

psis

Categorization of unexpected events during treatment and transport (TBD)	
An categorized evaluation of the level of the receiving hospitals response on arrival (1 = inadequate; 2 = adequate; 3 = hyper-respo	
Categorization of equal medical alternatives given the availability (TBD)	
Evaluation of mission content versus activation call from dispatch center (urgency)	
	Requires that each unit has defined the minimal standard of care in a given diagnosis
n of follow-up studies measuring ir	The Eq-5d is simple to obtain

in emergency medicine, Specialist in surgery, in training for speciality in surgery, Other type of physician (specify)

ard, ER, Other (specify)
cult airway managment
other (specify)

equipment, Syringe-pumps (1,2,3,more), BIS, Continous capnography, nose-sampling capnography, Blood-sugar measurment equipmen

itants.

ICU patient from higher to lower level of treatment, transfer of other patient to higher level of treatment, transfer of patient between nati

police, necessary medical treatment to make patient transportable or to improve prognosis

ation, pacing etc) 9 cardiac massage 10 tracheostomy 11 other

›nse; 4 = not evaluated)

t, Pressors, Anesthetics, trombolytic drugs....++

ions for special type of care, transfer of neonatal patient in incubator to higher level of care, transfer of neonatal patient in incubator to lo

ver level of care
