# Supplementary material for: A consensus-based template for documenting and reporting in physician-staffed pre-hospital services
Source: Scand J Trauma Resusc Emerg Med. 2011 Nov 23;19:71. doi: 10.1186/1757-7241-19-71 (PMC3282653; doi:10.1186/1757-7241-19-71)
Supplement: Additional file 2 — Results Stage 2. All rankings from experts on proposals from stage 1 [file 1757-7241-19-71-S2.PDF]

The Utstein Template for documenting and reporting in physician-staffed pre-hospital services.

Defining a minimum core data set for a common European standard.

AIM: To establish a common core data set with definitions for activity documentation and shared research efforts. Core variables should be pos

Ranking- second round

In the spreadsheet below the proposed data points from the experts are presented. At the left collumn

In the red collumn you should list your top 10 data points using numbers from 10-1. The value 10 mea

Use all numbers only once within each section (Fixed system variables-Event operational descriptors-pa

Data points marked in blue is proposed as optional. To give points to optional data points is allowed, bu

Fixed system variables.

Definition: Variables crucial for comparisons between services and/or countries. Ask yourself: "what would I like to know if I were

No of expert proposal	Expert 1	Expert 2	Expert 3	Expert 4
12	10		1	10
6	9	1		4
5	7		2	7
5	6			6
5	8	5	6	9
3	5	10	9	8
2	3	7		
2	4			

2				
1	2		5	3
1				
1				
1		4		
1			4	
1	1			
1			3	
1		6	7	1
1		9		
1		3		
1				
1		2		
1				
1		6		
1				
1				
1		8	8	5
1				
1				
1				2
1				
1			10	
1				

Event operational descriptors

Definition: Variables related to indication for dispatch, timelines for event and logistics.

No of expert proposal	Your ranking from 10-1	Your ranking from 10-1	Your ranking from 10-1	Your ranking from 10-1
13	10	10	1	8
11	9	9		9
6	8			10
5	7			7
4	3			4
4			2	

4	6		3	5
3				6
3		7	5	
3		6	6	
3		5	7	
3	5			2
3	1			1
2	4			
2				
1				
1	2	4		3
1		3		
1				
1				
1				
1				
1				
1				
1			10	
1		8	9	
1				
1				
1			8	
1		2		
1				
1				
1		1		
1				
1				
1				

Patient descriptors

Definition: Patient characteristics such as age, gender, co-morbidity eg.

No of expert proposal	Your ranking from 10-1	Your ranking from 10-1	Your ranking from 10-1	Your ranking from 10-1

11	10		3	10
10	9	2	1	5
10	8	1	2	4
6	7			9
6	2	6		
5	3		4	8
3			5	7
3	6	4	6	6
2	5	3	7	
2	1			3
2		9		
2		5		
1				
1				
1				
1	4		10	
1				
1		7	1	
1				
1				
1				
2		8		
2			9	
2				
2			8	
2		10		
2				
1				2
1				
1				
1				
1				
1				1

Process mapping

Definition: Variables related to what happened to the patient, such as treatments and procedures performed.

No of expert proposal	Your ranking from 10-1	Your ranking from 10-1	Your ranking from 10-1	Your ranking from 10-1
14	10	10		10
7	9	8		8
5	8	7		9
4	1			
4	6		5	3
4	5			6
3		5		
3		4		
2				
2		2		
2	7			5
2	4	9		
2	3	1		4
1				
1			4	
1				
1				
1				
1	2			2
1				
1				
1		6	1	
1			2	
1			3	
1				7
1				
2				
2				
1				
1		3		
1				1

1				
1				
1				
1				
1				
1				

Outcome measures or Quality Indicators- Optional

Definition: Suggest any outcome measures or quality indicators during the pre-hospital phase of care.

No of expert proposal	Your ranking from 10-1	Your ranking from 10-1	Your ranking from 10-1	Your ranking from 10-1
4	10	10	2	10
3		9		
2		8		
2	7	5		9
2	5			
2		1	1	8
1				6
1	9			5
1				7
1				
1				4
1				
1				
1				
1			4	
1			3	
1				
1	4	4		
1	8	3		
1		2		
1				
1	6	6		
1				
1		7		

1	3			3
1	1			2
1				
1				1
1	2			

ssible to collect routinely, and should be easy to adapt to most excisting softwares.

ı the number of experts proposing the data points are listed.

ns greatest importance/relevance, and 1 less important.
ıtient descriptors- Process mapping- Outcome Indicators)

ıt pay attention to results from first round.

ː to compare my results with another service?"

Expert 5	Expert 6	Expert 7	Expert 8
6	3	10	8
5	7	8	1
7		9	7
9	8	7	9
1	4	5	
10		6	10
		3	6

8	5	1	
2	6		
			3
3	2		
			5
	1		
	9	3	4
			1
		4	
4	10	4	
		2	
		6	2

Your ranking from 10-1	Your ranking from 10-1	Your ranking from 10-1	Your ranking from 10-1
	1		7
10	2	7	8
9	4	10	9
3	3	9	10
7		3	
2			2

8		1	
			6
4		8	
			3
5	9	5	5
		3	2
	10	6	
1			4
	5	4	
	6	4	
	7	4	
		4	
			1
	8		
		2	
6			

**Your ranking from 10-1**	**Your ranking from 10-1**	**Your ranking from 10-1**	**Your ranking from 10-1**

10	7	7	10
9	1	9	7
8	2	8	6
5		3	8
7	3	5	9
6	4	10	5
4			4
2		1	
3	10	6	3
	5		2
	9		
	6		
	8		1
		2	
		5	
		4	

Your ranking from 10-1	Your ranking from 10-1	Your ranking from 10-1	Your ranking from 10-1
10	1	10	10
7		7	
8	8	6	9
	2		
9	3	8	8
	4	5	9
5	9	9	
	5		7
	6	4	
4			
		1	
			1
	7		5
6	10		4
		3	3
			2
3		2	

			6

Your ranking from 10-1	Your ranking from 10-1	Your ranking from 10-1	Your ranking from 10-1
10		1	
	1		9
9	2		10
	3	9	
	8		
8			1
			4
	9		
	4		2
	5		
			7
			6
	7	10	
7	6	8	8
		7	
		6	
			3
			5

		5	
		4	
		2	
		3	

Expert 9	Expert 10	Expert 11	Expert 12
9	1	10	8
		1	7
10	2		9
6		9	10
5	6		
3	3		2
			3
	4		

	7	8	
	5	7	1
8			
			5
1		2	4
4			6
	8		
2			
	10		
7			
		4	
	9		
		5	
		6	

Your ranking from 10-1	Your ranking from 10-1	Your ranking from 10-1	Your ranking from 10-1
10		9	9
9		3	
8	1	7	6
7	2	8	1
6			8

2	4		2
	3		
5	5		
	6	6	
	6	5	
3	7		
		10	
1	9	2	3
	10	1	4
			5
	8		
4			7
			10
		4	

Your ranking from 10-1	Your ranking from 10-1	Your ranking from 10-1	Your ranking from 10-1

10		8	1
9	1	10	2
8	2	9	3
7	5	7	8
6		5	
		6	4
5			
4			
3	9		10
			9
		4	
	4		
		3	
			7
2	6	2	
	7		
	3		
		1	
	10		
			5
1			
	8		
			6

Your ranking from 10-1	Your ranking from 10-1	Your ranking from 10-1	Your ranking from 10-1
10	1	10	1
9			5
8			6
7		8	7
1			8
	2		
		7	4
	3		
		9	
3	4		
4			
5	5		
2	6		
6			
		2	
		1	
		4	3
	8		
	9		
			9

	10		
		6	2
		5	
		3	10
	7		

Your ranking from 10-1	Your ranking from 10-1	Your ranking from 10-1	Your ranking from 10-1
			2
		3	
6	1	2	
8	2		3
10			
			4
7			5
	3		
	4	8	
			1
			6
		9	
2	-5		
9	-6	5	7
		4	8
5			
4	7		9
	8	7	
		10	

1	10	6	
	9		
3		1	

Expert 13	Expert 14	Data point	Core / optional
9	10	Educational level physician	Core
	8	Operating hours	Core
	7	Crew composition	Core
7	5	Population	Core
10		mode of transportation	core
6	4	Mission types	core
		tier response	core
		median response time ambulance	core

	6	Service Area	Core
5		Number of responses/missions per year	core
		Non MD-ALS unit hours per 100,000	core
1		M,W (s), Z statistic	Core
		In-hospital training	Core
		MD-ALS unit hours (service area)	core
		Training level	Core
4		MD-ALS unit hours per 100,000	core
8	3	Response time	Core
		Intubation rate	Core
		Rescue system	core
		fixed base	core
		Funding of service	core
		Equipment	Core
		dispatch system	core
		distance/time to trauma center	core
3	9	Annual nb of responses	Optional
2	2	Activation criteria	Optional
		Non MD-ALS unit hours (service area)	optional
		Type of hospitals in coverage area	Optional
	1	Categorization of missions	Optional
		Percentage of runs aborted en route	Optional
		Percentage of physician assisted runs in relatior	Optional
		experience of physician in HEMS	optional

Your ranking from 10-1	Your ranking from 10-1	Data point	Core/optional
	9	Date and time of call	core
	10	Type of dispatch	Core
10	8	Time from alarm to arrival at scene	core
9	4	On scene time	Core
	5	Reason for aborted mission	Core
	10	dispatch code (level of acuity)	core

Your ranking from 10-1	Your ranking from 10-1	Data point	Core/optional
8		type of transportation	core
	6	Response time	Core
		arrival on scene	core
		departure from scene	core
		Arrival at receiving facility	Core
5	7	Transport time	Core
7		type of destination hospital	core
		Mission completion	Core
		type of response	core
		Delayed	Core
6		Specific reasons for prolonged on scene time?	Core
	2	Other resource on site +/- 5 minutes of your ar	Core
		Total time in service	Core
4		Trauma	core
3		Medical	core
2		Paediatric	core
		Obstetric/gynecol.	core
		time logistics (utstein template dispatch)	core
1		Geographic data	core
		verified code	optional
		Time from alarm to hospital arrival	optional
		Means of transport to scene	optional
		Patients treated by physician	optional
		Total number of units dispatched	optional
		Highest level of prehospital care provider (Befo	Optional
		Unit mobile	Optional
	3	Type of transportation	Optional
		Arrival at the scene	Optional
		Departure from scene	Optional
		Arrival at hospital	Optional

	3	Co-morbidity	core
9	10	age	core
8	9	gender	core
	5	RTS delta/MEES	core
7	8	GCS	Core
10	7	Medical problem (main reason for response):	Core
6		Injury Severity Score	core
5	1	Patient Category	Core
4		mechanism of injury	core
		NACA	Core
2		Heart rate and rythm	core
3		pain assesment	core
		In trauma: position in vehicle	Core
		In medical: situation of patient	Core
	7	Surgical problem (main reason for respone)	Core
		cardiac arrest	core
	6	condition when met compared to alarm informa	core
1		BP(systolic)	core
		social situation	core
		Survival status upon leaving patient	core
		Drug-abuse	Core
		SpO2	optional
		SBP on arrival	optional
		RTS on admission	Optional
		GCS on arrival	optional
		Respiratory Rate	Optional
		Dominating type of injury	Optional
	4	Vital data before and after treatment	optional
		AIS region(s) with score>2	optional
		GCS on admission	Optional
		HR categories	optional
	2	prehospital airway management	optional
		Dominationg type of medical incident	Optional

**Your ranking from 10-1**	**Your ranking from 10-1**	**Data point**	**Core / optional**
10	9	Airway management	core
2		Surgical intervention	Core
1		Diagnostic intervention	Core
		iv access	core
9	7	Medication	Core
		CPR	Core
		Vascular intervention	Core
		i.o. access	core
8	5	In CA: CPR started by bystander?	Core
		blood test on scene	core
		Other intervention	Core
7	6	ventilation	core
		Monitoring	core
		Other persons at site?	Core
		Basic medical help provided by bystander?	Core
4		IN CA: Airway secured by other EMS unit?	Core
	1	Diagnostic importance of physician?	Core
		Consequence: changed admittance, changed or	Core
	3	Theraperutic importance of phsysician	Core
		If yes: could treatment have been provided by (	Core
	4	trombolysis?	core
	8	procedures	core
6	10	immediate outcome	core
5		late outcome	core
		Non-invasive interventions	core
	2	prehospital care at all	core
		Thoracic drainage	Optional
3		Ventilation	optional
		Immobilised	Optional
		Hemostasis	Optional
		Incubator	Optional

		CPR	Optional
		If yes: type of airway management	Optional
		Ultrasound diagnostics	Optional
		Blood sample on scene	Optional
		Enrollment in scientific protocol	Optional
		Adjuncts	optional

Your ranking from 10-1	Your ranking from 10-1	Data point	Core / optional
8	9	Delta MEES	
	8	Delta GCS	
	7	Delta RTS	
9		Glasgow Outcome Scale	
10		LOS in-hospital	
7	6	Precision of dispatch code (retrospectively)	
		W statistic	
	10	HEMS benefit score	
		need for transport	
		NACA upon arrival of EMS personnel at scene	
6		VAS (visual analogue pain score) at arrival and at arrival in hospital	
		BP (MAP) at arrival and at arrival in hospital	
		HR, RF at arrival and at arrival in hospital	
		number of procedures before succes (above)	
		first unit dispatched= highest level unit on scene	
4	4	hospital of arrival= hospital of definitive treatment	
		Any intended procedure not carried out	
		Discharge destination	
3	5	Survival status	
		Final diagnosis	
2		Abbreviated Injury Scale (AIS)	
5		ICU-Time	
	9	EtCo2, SpO2, RR, HR, SBP before and after treatment/management	
		Adherence to treatment protocols in any given patient	

		Complication	
	3	Hospital response	
		Valid alternatives	
		Validy of activation	
1		Quality of life	

**Variable categories**	**Exact definition of data point**
Predfined string: Type of education/speciality	Specialist in anaesthesiology, in training for spe
Predefined string: When is your pysician-staffed service operational?	24/7, all week day and evening, all week only d
Predfined string: When performing ALS or other advanced effort: who is	HEMS Paramedic, HEMS anesthetic nurse, HEMS
Number	
1 = ground ambulance<br>2 = helicopter ambulance<br>3 = fixed-wing ambulance<br>4 = not transported<br>5 = unknown	Main type of transportation vehicle (if multiple chose vehicle used for the majority of the transportation phase)
trauma/internal/mix of missions, OB-GYN, newborn, interhospital transfer	

Squared kilometres	
	as from Unit hours ALS 'European Emergency D
Number: Number of months per year	% of full time work
	Annual unit hours of physician ALS per km2 of s
Number: Number of years in pre-hospital service	Months (full time work)
	as from Unit hours ALS 'European Emergency D
1 = fixed system, 2 = Rendez-vous system	1 = Physician and Paramedics use the same vel
yes/no	
state/commercial/private funded	
Predfined string: types of equipment	Ventilator (advanced), ventilator (simple), Defib
alarm center/special HEMS center/	
Continuous	Number of activated responses annually
1 = criteria based; 2 = consultation w/ physician; 3 = both	Description of how decision of response is taken
	as above
1 = Primary; 2 = Inter-hospital; 3 = SAR; 4 = Other	Categorization of different types of mission with
yr	

**Variable categories**	**Exact definition of data point**
Predefined string:	Emergency medical mission, emergency trauma
	The net driving (flying) tim to patient site
Number (minutes):	The net time from reashinbg patient to start of
Predefined string:	Weather, other higher priority mission, updated

**Variable categories**	**Exact definition of data point**
Number (minutes):	Time from alarm to initiation om mission
Hours and minutes	Time when unit arrives at emergency departme
Number (minutes):	The net driving (flying) time to hospital
1-3 = Level 1-3	
Predefined string:	Completed mission, Aborted mission, Mission ha
(i.e. ambulatory care, transport without physicians, transport with physician, air lift, dead on scene)	
Yes/No:	Yes: reason, No: reason
Predfined string:	Lack of resources, entrapped patient, difficulty
Predfined string:	Police, Firedep, Security, Social care, Home nur
	In the event of ambulance run aborted en route
1 = traffic, 2 = occupational, 3 = leisure/sports, 4 = other	
1 = ACS, 2 = stroke, 3 = other cardiovascular, 4 = airway & breathing, 5 = seizure, 6 = unconsciousness, 7 = endocrino	
1 = airway/breathing, 2 = cardiovasc., 3 = seizure, 4 = infectious, 5 = other	
1 = bleeding, 2 = eclampsia, 3 = pregnancy other	
times	
location of the emergency (i.e. public place, road, highway)	
	priority as judged by physician on scene
	as from Utstein trauma registry
1 ground ambulance 2 car 3 helicopter 4 other	The type of vehicle that transported the physici
1 one 2 two 3 > two	
	the number of rescue units dispatched to the ev
Hours and minutes	Time when unit is mobile
Nominal	Main type of transportation vehicle (if multiple;

Pre event ASA classification	
Revised Trauma Score/ Mainz Emergency Evaluation Scoring	
ICD-10	X.XX
Blunt trauma/penetrating trauma/non-trauma (incl drownings and burns/unknown	
as from Utstein Trauma Registry + burns and drowning	
Ordinal (1-7) according to NACA	Classification of the medical severity (on-scene
SR/FA/SVT/etc	
	VAS score
String (predfined):	Driver, passenger front, backseast right, backse
String (predfined):	Home, stairs, elevator, pavement, other outdoo
ICD-10	X.XX
1 yes on arrival 2 yes after arrival 3 never	whether CA occurred at any time before arrival
same/worse/better	
(i.e. homeless, deprivation, criminal background...)	
	If pertinent (Excluding missions without any pa
String (predfined):	Herione, Other opioid, Cocaine, Amphetamine, (
Ordinal (according to Utstein Trauma Registry)	Revised Trauma Score (RTS) categories with cli

AIS regions	
Eye-, verbal- and motor-score	GCS on admission and/or after necessary interv
1 = <100, 2 = >100	

Variable categories	Exact definition of data point
According to Utstein Airway	
Nominal	1 = thoracostomy (incl needle-decompression);
Nominal	1 = Ultrasound; 2 = Invasive pressure monitori
analg/cardiac/sedation/inotropic/etc	According to ATC (4th level)
Nominal	Registration according to Utstein template for c
Nominal	1 = volume replacement (TBD); 2 = compressiv
Y/N	
Nominal	1 = CPR; 2 = defibrillation; 3 = cardioversion;
spont//CPAP/hand assist/mech	
1 = BP, 2 = pulse oximetry, 3 = ECG, 4 = capnography	
Y/N	
Y/N	
Y/N	
Y/N	
Y/N	
Y/N	
Y/N	
1 IV line 2a IV drugs 2b fibrinolysis 3 nebulization 4 bag mask ventilation 5 supraglottic device 6 intubation & ventilation	
1 dead on scene-no treatment 2 dead on scene after treatment 3 transported alive to hospital	
1 alive 2 dead	outcome at 30 days
1 = iv access, 2 = io access, 3 = iv/io drug, 4 = oxygen spont. breathing, 5 = BVMV, 6 = supraglottic device	
1 = manual BV, 2 = ventilator	

String (predfined):	
1 = telemetric ECG-transmission	

Variable categories	Exact definition of data point
physiological scoring before and after intervention HR, RF, Pain, GCS, SBT	
worst before intervention vs. best after intervention	
at arrival and at arrival in hospital	
5 = Good Recovery; 4 = Moderate disability; 3 = Severe disability; 2 = F	Glasgow Outcome Scale – at discharge from ma
Number	days
1 yes 2 no, verified higher 3 no, verified lower	
	J Trauma. 2005 Jun;58(6):1272-6; discussion 1
0-8	
yes/no	
worst before intervention vs. best after intervention	
1 yes 2 no	
1 yes 2 no	
1 = Home; 2 = Rehab; 3 = Morgue; 4 = Higher treatment level; 5 = And	The patient's destination after end of acute care
1 = Dead; 2 = Alive; 3 = Unknown	Alive or dead 30 days after event
Ordinal	Diagnosis on discharge according to ICD-10 (co
Ordinal	AIS severity codes that reflect the injuries
Number	days

To be decided	Categorization of unexpected events during trea
To be decided	An categorized evaluation of the level of the rec
To be decided	Categorization of equal medical alternatives giv
To be decided	Evaluation of mission content versus activation
EQ-5D (and HUI)	

Comments for discussion
ecialty in anaesthesiology, Specialist in emergency medicine, in training for speciality in emergency medicine, Specialist in surgery, in trainir
laytime, working days day and night, working days 24h, working days only daytime, other (specify)
It is improtant to control all variables. The assistant is important - especially in unanticipated difficult airway managment
is the doc car called out at once or later ?
To get an impression on the system

ata project': Annual unit hours of Non-ALS (physician only in this case)hours per 100,000 inhabitants.

important to register to what degree the physicians maintain procedures with in-hospital training

service area

ata project': Annual unit hours of ALS (physician only in this case) per 100,000 inhabitants.

nicle for patient approach

orillator, Invasive BP-measurment tool, 12-lead ECG, 3 or 5 lead ECG, NO-inhalation equipment, Syringe-pumps (1,2,3,more), BIS, Continou

Definition

n

Description of different types

Comments for discussion

a mission, Transfer of ICU patient from lower to higher level of treatment, transfer of ICU patient from higher to lower level of treatment, tra

transport

l infor of no need for responce

nt or hospital and/or transfer of treatment responsibility

anded over to other unit

evacuating patient from site to waiting ambulance, threats, need for assistance from police, necessary medical treatment to make patient tra

se, GP, MD passing by, Other health care proffessional passing by

[illegible]

ology, 8 = other

alternatively, the NACA score. It can also be used to cross-check the accuracy of dispatch

an to the place of event

vent, including the one transporting the physician

See trauma utstein

type used for majority of trsp phase)

Core variable if pertinent

Core variable if pertinent

Core variable if pertinent

Comments for discussion

See Utsein Airway
Decision of scale and levels
east left, backseast middle
r (define)
at hospital
tient contact)
Other sentral stimulating drug, Cannabis, Benzodiazepines, Ither (define).
Description of sedated/intubated pts
See trauma utstein
Description of sedated/intubated pts

**Comments for discussion**
Decision on nb of interventions
Decision on nb of interventions
Decision on level of registration
ardiac arrest and CPR
Decision on nb of interventions
Decision on nb of interventions
7 chest drain 8 electric stimulation of the heart (includes defibrillation, pacing etc) 9 cardiac massage 10 tracheostomy 11 other

Comments for discussion

ain hospital

1277.

I.E: Intended intubation not possible.

e in the initial (main) hospital

mpared with tentative diagnosis)

Requires that each unit has defined the minimal standard of care in a given diagnosis

atment and transport (TBD)
ceiving hospitals response on arrival (1 = inadequate; 2 = adequate; 3 = hyper-response; 4 = not evaluated)
en the availability (TBD)
call from dispatch center (urgency)
The Eq-5d is simple to obtain

ıg for speciality in surgery, Other type of physician (specify)

us capnography, nose-sampling capnography, Blood-sugar measurment equipment, Pressors, Anesthetics, trombolytic drugs....++

nsfer of other patient to higher level of treatment, transfer of patient between nations for special type of care, transfer of neonatal patient in inc

ansportable or to improve prognosis

cubator to higher level of care, transfer of neonatal patient in incubator to lover level of care
