# Supplementary material for: A consensus-based template for documenting and reporting in physician-staffed pre-hospital services
Source: Scand J Trauma Resusc Emerg Med. 2011 Nov 23;19:71. doi: 10.1186/1757-7241-19-71 (PMC3282653; doi:10.1186/1757-7241-19-71)
Supplement: Additional file 3 — Proposals before consensus meeting. All proposals organized by the sum of ranking points from stage 2. [file 1757-7241-19-71-S3.PDF]

The Utstein Template for documenting and reporting in physician-staffed pre-hospital services.

Defining a minimum core data set for a common European standard.

AIM: To establish a common core data set with definitions for activity documentation and shared research efforts. Core variables should be pos

**Fixed system variables.**				
**Definition: Variables crucial for comparisons between services and/or countries. Ask yourself: "what would I like to know if I were**				
**No of expert proposal**	**Expert no 1**	**Expert no 2**	**Expert no 3**	**Expert no 4**
12	10		10	10
5	6			6
5	7		9	7
3	5	10	2	8
5	8	5	5	9
6	9	1		4
1		8	3	5
1		6	4	1
1	2		6	3
2				
1				
1		9		
1				2
1			8	
1		6		
2	4			
2	3	7		
1			7	
1				

1			1	
1		4		
1	1			
1				
1				
1		3		
1		2		
1				
1				
1				
1				
1				
1				
**Event operational descriptors**				
**Definition: Variables related to indication for dispatch, timelines for event and logistics.**				
**No of expert proposal**	**Your ranking from 10-1**	**Your ranking from 10-1**	**Your ranking from 10-1**	**Your ranking from 10-1**
13	10	10	10	8
11	9	9		9
4	6		8	5
4	3			4
1				
2				
1				
3		7	6	

1				
1				
1				

Patient descriptors

Definition: Patient characteristics such as age, gender, co-morbidity eg.

No of expert proposal	Your ranking from 10-1	Your ranking from 10-1	Your ranking from 10-1	Your ranking from 10-1
10	9	2	10	5
10	8	1	9	4
11	10		8	10
5	3		7	8
6	7			9
6	2	6		
2	5	3	4	
2	1			3
3			6	7
3	6	4	5	6
2		8		
2		9		

2		5		
1	4		1	
2			3	
1		7		
2		10		
1				2
2				
2			2	
1				
1				
1				
1				
1				
2				
1				1
1				
1				
1				
1				
1				

**Process mapping**				
**Definition: Variables related to what happened to the patient, such as treatments and procedures performed.**				
**No of expert proposal**	**Your ranking from 10-1**	**Your ranking from 10-1**	**Your ranking from 10-1**	**Your ranking from 10-1**
14	10	10		10
5	8	7		9
4	6		6	3
7	9	8		8
1			9	
2	4	9		
1		6	10	
4	5			6
3		5		
2				

4	1			
2	7			5
1				
1			8	
2	3	1		4
1				
3		4		
1				
1	2			2
1				
1		3		
2				
1				7
1				
1				
1			7	
1				
2				
1				
1				
1				
1				
2		2		
1				
1				1
1				
1				

**Outcome measures or Quality Indicators- Optional**				
**Definition: Suggest any outcome measures or quality indicators during the pre-hospital phase of care.**				
**No of expert proposal**	**Your ranking from 10-1**	**Your ranking from 10-1**	**Your ranking from 10-1**	**Your ranking from 10-1**
4	10	10	9	10
1	8	3		
2	7	5		9

2		1	10	8
1	6	6		
2		8		
2	5			
3		9		
1				
1	3			3
1				4
1	9			5
1			8	
1	4	4		
1		2		
1				6
1				7
1	1			2
1				
1		7		
1				
1				
1				
1			7	
1	2			
1				
1				1
1				
1				

ssible to collect routinely, and should be easy to adapt to most excisting softwares.

to compare my results with another service?"			
**Expert no 5**	**Expert no 6**	**Expert no 7**	**Expert no 8**
6	3	10	8
9	8	7	9
7		9	7
10		6	10
1	4	5	
5	7	8	1
4	10	4	
	9	3	4
2	6		
8	5	1	
			1
		6	2
	1		
		4	
		3	6
3	2		

			3
			5
		2	
**Your ranking from 10-1**	**Your ranking from 10-1**	**Your ranking from 10-1**	**Your ranking from 10-1**
	1		7
10	2	7	8
8		1	
7		3	
	6	4	
	10	6	
	7	4	

		2	
**Your ranking from 10-1**	**Your ranking from 10-1**	**Your ranking from 10-1**	**Your ranking from 10-1**
9	1	9	7
8	2	8	6
10	7	7	10
6	4	10	5
5		3	8
7	3	5	9
2		1	
3	10	6	3
4			4
	8		1
	5		2

	9		
	6		
		5	
		4	
		2	

**Your ranking from 10-1**	**Your ranking from 10-1**	**Your ranking from 10-1**	**Your ranking from 10-1**
10	1	10	10
8	8	6	9
9	3	8	8
7		7	
3		2	
	4	5	9
5	9	9	
	5		7

	2		
	6	4	
6	10		4
4			
			6
	7		5
		3	3
		1	
			1
			2
**Your ranking from 10-1**	**Your ranking from 10-1**	**Your ranking from 10-1**	**Your ranking from 10-1**
10		1	
7	6	8	8
	3	9	

8			1
			3
9	2		10
	8		
	1		9
			5
		5	
	5		
	7	10	
		7	
			4
	9		
		4	
			6
		6	
			7
	4		2
		3	
		2	

**Expert no 9**	**Expert no 10**	**Expert no 11**	**Expert no 12**
9	1	10	8
6		9	10
10	2		9
3	3		2
5	6		
		1	7
7			
1		2	4
	5	7	1
	7	8	
	10		
4			6
	9		
			5
	8		
	4		
			3
8			

		6	
		4	
		5	
2			
**Your ranking from 10-1**	**Your ranking from 10-1**	**Your ranking from 10-1**	**Your ranking from 10-1**
10		9	9
9		3	
2	4		2
6			8
	10	1	4
	6	5	
			5

4			7
		10	
**Your ranking from 10-1**	**Your ranking from 10-1**	**Your ranking from 10-1**	**Your ranking from 10-1**
9	1	10	2
8	2	9	3
10		8	1
		6	4
7	5	7	8
6		5	
3	9		10
			9
5			
4			
2	6	2	
		4	

	4		
	3		
		3	
		1	
			5
	10		
	7		
			6
	8		
			7
1			

**Your ranking from 10-1**	**Your ranking from 10-1**	**Your ranking from 10-1**	**Your ranking from 10-1**
10	1	10	1
8			6
1			8
9			5
2	6		
		9	
5	5		
	2		
	3		

7		8	7
6			
3	4		
	10		
		7	4
		3	10
			9
	8		
		2	
	9		
4			
		6	2
		4	3
	7		
		5	
		1	

**Your ranking from 10-1**	**Your ranking from 10-1**	**Your ranking from 10-1**	**Your ranking from 10-1**
			2
9	-6	5	7
6	1	2	

4	7		9
8	2		3
		3	
	8	7	
1	10	6	
7			5
			6
2	-5		
		4	8
10			
			4
	9		
	4	8	
		10	
5			
	3		
		9	
3		1	
			1

**Expert no 13**	**Expert no 14**		**Data point**
9	10	104	Service Area
7	5	82	Population
	7	74	Crew composition
6	4	69	Mission types
10		58	mode of transportation
	8	51	Operating hours
2	2	45	Activation criteria
8	3	45	Response time
5		37	Number of responses/missions per year
	6	35	Service Area
3	9	22	Annual nb of responses
		20	Intubation rate
	1	20	Categorization of missions
4		18	MD-ALS unit hours per 100,000
		18	dispatch system
		17	median response time ambulance
		13	tier response
		12	MD-ALS unit hours (service area)
1		9	M,W (s), Z statistic

		7	Percentage of physician assisted runs in relatio
		7	In-hospital training
		6	Training level
		6	Type of hospitals in coverage area
		5	Percentage of runs aborted en route
		3	Rescue system
		2	Funding of service
		2	distance/time to trauma center
			Non MD-ALS unit hours per 100,000
			fixed base
			Equipment
			Non MD-ALS unit hours (service area)
			experience of physician in HEMS
**Your ranking from 10-1**	**Your ranking from 10-1**		**Data point**
	9	83	**Mission time**
	10	76	**Type of dispatch**
8		44	**type of transportation**
	5	36	**Mission outcome**
3		28	Medical
		27	type of response
2		18	Paediatric
		13	arrival on scene

		11	Time from alarm to hospital arrival
		10	Total time in service
		2	Means of transport to scene
**Your ranking from 10-1**	**Your ranking from 10-1**		**Data point**
9	10	93	**age**
8	9	85	**gender**
	3	84	**Co-morbidity**
10	7	70	Medical problem (main reason for response):
	5	64	RTS delta/MEES
7	8	58	**GCS, HR, Syst blpr, ecg,rr,spO2,pain**
4		41	mechanism of injury
		35	NACA
6		32	Injury Severity Score
5	1	31	Patient Category
		27	SpO2
2		22	Heart rate and rythm

3		17	pain assesment
		15	cardiac arrest
		11	GCS on arrival
1		11	BP(systolic)
		11	Respiratory Rate
	4	11	Vital data before and after treatment
		10	Dominating type of injury
		9	SBP on arrival
	2	8	prehospital airway management
		8	HR categories
		7	Survival status upon leaving patient
	6	6	condition when met compared to alarm informa
		4	GCS on admission
		2	RTS on admission
		1	Dominationg type of medical incident
		1	AIS region(s) with score>2
		0	In trauma: position in vehicle
		0	In medical: situation of patient
		0	social situation
		0	Drug-abuse

**Your ranking from 10-1**	**Your ranking from 10-1**		**Data point**
10	9	102	Airway management
1		70	Diagnostic intervention
9	7	68	Medication
2		55	Surgical intervention
6	10	38	immediate outcome
7	6	35	ventilation
	8	34	procedures
		31	CPR
		28	Vascular intervention
8	5	28	In CA: CPR started by bystander?

		25	iv access
		22	Other intervention
		20	Consequence: changed admittance, changed or
5		19	late outcome
		19	Monitoring
		16	If yes: type of airway management
		15	i.o. access
	1	13	Diagnostic importance of physician?
	3	13	Theraperutic importance of phsysician
		13	Enrollment in scientific protocol
		12	Hemostasis
3		11	Ventilation
		9	Non-invasive interventions
		9	Immobilised
	4	8	trombolysis?
		8	Basic medical help provided by bystander?
		8	Ultrasound diagnostics
		7	Thoracic drainage
		7	Adjuncts
4		5	IN CA: Airway secured by other EMS unit?
		5	Blood sample on scene
	2	3	prehospital care at all
		2	blood test on scene
		2	If yes: could treatment have been provided by o
		1	Incubator
			Other persons at site?
			CPR

**Your ranking from 10-1**	**Your ranking from 10-1**		**Data point**
8	9	69	Delta MEES
3	5	63	Survival status
9		51	Glasgow Outcome Scale

7	6	41	Precision of dispatch code (retrospectively)
5		40	ICU-Time
	7	36	Delta RTS
10		36	LOS in-hospital
	8	30	Delta GCS
	9	29	EtCo2, SpO2, RR, HR, SBP before and after trea
		28	Complication
6		27	VAS (visual analogue pain score) at arrival and
	10	24	HEMS benefit score
4	4	22	hospital of arrival= hospital of definitive treatm
		22	Discharge destination
		21	Final diagnosis
		20	W statistic
		20	need for transport
	3	19	Hospital response
		18	HR, RF at arrival and at arrival in hospital
		17	Adherence to treatment protocols in any given
2		13	Abbreviated Injury Scale (AIS)
		10	BP (MAP) at arrival and at arrival in hospital
		9	Any intended procedure not carried out
		7	first unit dispatched= highest level unit on scen
1		7	Quality of life
		6	NACA upon arrival of EMS personnel at scene
		4	Validy of activation
		2	Valid alternatives
		1	number of procedures before succes (above)

	Core / optional
	Core
	Core
	Core
	core
	core
	Core
	Optional
	Core
	core
	Core
	Optional
	Core
	Optional
	core
	core
	core
	core
	core
	Core

ı to total ambulance runs	Optional
	Core
	Core
	Optional
	Optional
	core
	core
	core
	core
	core
	Core
	optional
	optional
**Group meeting 1**	**Core / optional**
defined as data variable 32 in utstein trauma, exept replace physician and at sce	**core**
As defined 4 times; call dispatch- unit arrival on-scene- patient leaving scene- a	Core
ground ambulance, helicopter, fixed-wing, other, no transportation as in no 27 ι	**core**
Patient attended, mission aborted weather, mission aborted technical, no need	**Core**
in 49	core
in 49	core
**out outcome measures**	core
**patient characteristics**	core

in 48	optional
in 48	Core
**in system characteristics**	optional
	**Core / optional**
**as defined in Utstein trauma**	core
as defined in Utstein trauma	core
	core
Needs to be discussed according to categorization. FHQ? Extended.	Core
out	core
first and last, raw values	Core
	core
out	Core
out	core
out	Core
out	optional
out	core

out	core
out	core
out	optional
out	core
out	Optional
out	optional
out	Optional
out	optional
out	optional
out	optional
moves to outcome section	core
out	core
out	Optional
out	Optional
out	Optional
out	optional
out	Core
out	Core
out	core
out	Core
	**Core / optional**
	core
	Core
	Core
	Core
	core
	core
	core
	Core
	Core
	Core

	core
	Core
new inititated treatment	Core
	core
	core
	Optional
	core
	Core
	Core
	Optional
	Optional
	optional
	core
	Optional
	core
	Core
	Optional
	Optional
	optional
	Core
	Optional
	core
	core
other resource within 10 minutes?	Core
	Optional
	Core
	Optional
	**Core / optional**

tment/management	
at arrival in hospital	
ent	
patient	
e	

**Variable categories**	**Exact definition of data point**
Predfined string: Type of education/speciality<br>Number	Specialist in anaesthesiology, in training for spe
Predfined string: When performing ALS or other advanced effort: who is	HEMS Paramedic, HEMS anesthetic nurse, HEMS
trauma/internal/mix of missions, OB-GYN, newborn, interhospital transfer	
1 = ground ambulance<br>2 = helicopter ambulance<br>3 = fixed-wing ambulance<br>4 = not transported<br>5 = unknown	Main type of transportation vehicle (if multiple chose vehicle used for the majority of the transportation phase)
Predefined string: When is your pysician-staffed service operational?	24/7, all week day and evening, all week only d
1 = criteria based; 2 = consultation w/ physician; 3 = both	Description of how decision of response is taken
Squared kilometres	
Continuous	Number of activated responses annually
1 = Primary; 2 = Inter-hospital; 3 = SAR; 4 = Other	Categorization of different types of mission with
	as from Unit hours ALS 'European Emergency D
alarm center/special HEMS center/	
	Annual unit hours of physician ALS per km2 of s

Number: Number of months per year	% of full time work
Number: Number of years in pre-hospital service	Months (full time work)
1 = fixed system, 2 = Rendez-vous system	1 = Physician and Paramedics use the same veh
state/commercial/private funded	
	as from Unit hours ALS 'European Emergency D
yes/no	
Predfined string: types of equipment	Ventilator (advanced), ventilator (simple), Defib
	as above
yr	
**Variable categories**	**Exact definition of data point**
define when does patient leave scene,	
Predefined string:	Emergency medical mission, emergency trauma
patient transportation	
Mission: unit dispatched but no patient contact.	Operational, other higher priority mission, upda
1 = ACS, 2 = stroke, 3 = other cardiovascular, 4 = airway & breathing, 5 = seizure, 6 = unconsciousness, 7 = endocrino	
(i.e. ambulatory care, transport without physicians, transport with physician, air lift, dead on scene)	
1 = airway/breathing, 2 = cardiovasc., 3 = seizure, 4 = infectious, 5 = other	

	as from Utstein trauma registry
	In the event of ambulance run aborted en route
1 ground ambulance 2 car 3 helicopter 4 other	The type of vehicle that transported the physici
**Variable categories**	**Exact definition of data point**

Pre event ASA classification	
ICD-10	X.XX
Revised Trauma Score/ Mainz Emergency Evaluation Scoring	
as from Utstein Trauma Registry + burns and drowning	
Ordinal (1-7) according to NACA	Classification of the medical severity (on-scene
Blunt trauma/penetrating trauma/non-trauma (incl drownings and burns/unknown	
SR/FA/SVT/etc	

	VAS score
1 yes on arrival 2 yes after arrival 3 never	whether CA occurred at any time before arrival

1 = <100, 2 = >100	
	If pertinent (Excluding missions without any pa
same/worse/better	
Eye-, verbal- and motor-score	GCS on admission and/or after necessary interv
Ordinal (according to Utstein Trauma Registry)	Revised Trauma Score (RTS) categories with cli
AIS regions	
String (predfined):	Driver, passenger front, backseast right, backse
String (predfined):	Home, stairs, elevator, pavement, other outdoo
(i.e. homeless, deprivation, criminal background...)	
String (predfined):	Herione, Other opioid, Cocaine, Amphetamine, (

**Variable categories**	**Exact definition of data point**
According to Utstein Airway	
Nominal	1 = Ultrasound; 2 = Invasive pressure monitori
analg/cardiac/sedation/inotropic/etc	According to ATC (4th level)
Nominal	1 = thoracostomy (incl needle-decompression);
1 dead on scene-no treatment 2 dead on scene after treatment 3 transported alive to hospital	
spont//CPAP/hand assist/mech	
1 IV line 2a IV drugs 2b fibrinolysis 3 nebulization 4 bag mask ventilation 5 supraglottic device 6 intubation & ventilation	
Nominal	Registration according to Utstein template for c
Nominal	1 = volume replacement (TBD); 2 = compressiv
Y/N	

Nominal	1 = CPR; 2 = defibrillation; 3 = cardioversion;
Y/N	
1 alive 2 dead	outcome at 30 days
1 = BP, 2 = pulse oximetry, 3 = ECG, 4 = capnography	
String (predfined):	
Y/N	
Y/N	
1 = manual BV, 2 = ventilator	
1 = iv access, 2 = io access, 3 = iv/io drug, 4 = oxygen spont. breathing, 5 = BVMV, 6 = supraglottic device	
Y/N	
1 = telemetric ECG-transmission	
Y/N	
Y/N	
Y/N	

**Variable categories**	**Exact definition of data point**
physiological scoring before and after intervention HR, RF, Pain, GCS, SBT	
1 = Dead; 2 = Alive; 3 = Unknown	Alive or dead 30 days after event
5 = Good Recovery; 4 = Moderate disability; 3 = Severe disability; 2 = P	Glasgow Outcome Scale – at discharge from ma

1 yes 2 no, verified higher 3 no, verified lower	
Number	days
at arrival and at arrival in hospital	
Number	days
worst before intervention vs. best after intervention	
To be decided	Categorization of unexpected events during trea
0-8	
1 yes 2 no	
1 = Home; 2 = Rehab; 3 = Morgue; 4 = Higher treatment level; 5 = Anc	The patient's destination after end of acute care
Ordinal	Diagnosis on discharge according to ICD-10 (co
	J Trauma. 2005 Jun;58(6):1272-6; discussion 1
yes/no	
To be decided	An categorized evaluation of the level of the rec
Ordinal	AIS severity codes that reflect the injuries
1 yes 2 no	
EQ-5D (and HUI)	
worst before intervention vs. best after intervention	
To be decided	Evaluation of mission content versus activation
To be decided	Categorization of equal medical alternatives giv

Comments for discussion

ecialty in anaesthesiology, Specialist in emergency medicine, in training for speciality in emergency medicine, Specialist in surgery, in trainir

It is improtant to control all variables. The assistant is important - especially in unanticipated difficult airway managment

laytime, working days day and night, working days 24h, working days only daytime, other (specify)

n

Definition

Description of different types

ata project': Annual unit hours of ALS (physician only in this case) per 100,000 inhabitants.

To get an impression on the system

is the doc car called out at once or later ?

service area

important to register to what degree the physicians maintain procedures with in-hospital training

icle for patient approach

ata project': Annual unit hours of Non-ALS (physician only in this case)hours per 100,000 inhabitants.

rillator, Invasive BP-measurment tool, 12-lead ECG, 3 or 5 lead ECG, NO-inhalation equipment, Syringe-pumps (1,2,3,more), BIS, Contino

Comments for discussion

mission, Transfer of ICU patient from lower to higher level of treatment, transfer of ICU patient from higher to lower level of treatment, tra

ted infor of not required

logy, 8 = other

an to the place of event

Comments for discussion

Decision of scale and levels

See Utsein Airway

at hospital
See trauma utstein
tient contact)
Description of sedated/intubated pts
Description of sedated/intubated pts
ast left, backseast middle
r (define)
Other sentral stimulating drug, Cannabis, Benzodiazepines, Ither (define).

**Comments for discussion**

Decision on nb of interventions
Decision on level of registration
Decision on nb of interventions
7 chest drain 8 electric stimulation of the heart (includes defibrillation, pacing etc) 9 cardiac massage 10 tracheostomy 11 other
ardiac arrest and CPR
Decision on nb of interventions

Decision on nb of interventions

**Comments for discussion**

ain hospital

atment and transport (TBD)

e in the initial (main) hospital

mpared with tentative diagnosis)

L277.

ceiving hospitals response on arrival (1 = inadequate; 2 = adequate; 3 = hyper-response; 4 = not evaluated)

Requires that each unit has defined the minimal standard of care in a given diagnosis

I.E: Intended intubation not possible.

The Eq-5d is simple to obtain

call from dispatch center (urgency)

en the availability (TBD)

ıg for speciality in surgery, Other type of physician (specify)

us capnography, nose-sampling capnography, Blood-sugar measurment equipment, Pressors, Anesthetics, trombolytic drugs....++

nsfer of other patient to higher level of treatment, transfer of patient between nations for special type of care, transfer of neonatal patient in inc

cubator to higher level of care, transfer of neonatal patient in incubator to lover level of care
